# Pharmaceuticals and radiopharmaceuticals in wastewater treatment plants: insights from an Arabian Peninsula nation

**DOI:** 10.1007/s11356-025-36287-6

**Published:** 2025-03-29

**Authors:** Ali Alfarsi, Anupama Kumar, Abbasher M. Gismelseed, Ahlam Al Azkawi, Marwa Al Mahdouri, Fadhila N. Al Mabsali, Sathish Babu, Yaqoob Al Harthy, Muna Al Hosni, Dayanthi Nugegoda

**Affiliations:** 1 RMIT University, Bundoora West Campus, Bundoora, VIC 3083 Australia; 2 CSIRO Environment, Waite Campus, Urrbrae, SA 5064 Australia; 3 Drug Safety Centre, Ministry of Health, Muscat, 100 Oman; 4 Department of Physics, College of Science, Sultan Qaboos University, Code 123, Al Khoud, P.O. Box 36, Muscat, Oman; 5 Central Analytical and Applied Research Unit (CAARU), College of Science, Sultan Qaboos University, Code 123, Al Khoud, Muscat, Oman; 6 Oman Water and Wastewater Services Company (OWWSC), Muscat, Oman

**Keywords:** Middle East and North Africa, Influent, Effluent, Contaminants of emerging concern, Sludge radioactivity, Pharmaceutical removal efficiency

## Abstract

The growing introduction of pharmaceutical compounds into waterways is a contemporary environmental issue in the Middle East and North Africa. This escalating problem necessitates a careful examination of the risks associated with the presence of pharmaceuticals in water sources. The present study analysed samples from eight wastewater treatment plants in an Arabian Peninsula nation. Sixty-nine percent of pharmaceuticals were partially or fully removed, while 31% showed higher effluent concentrations, indicating potential accumulation post-treatment. The highest detected pharmaceuticals were paracetamol (1300 ± 8 ng/L), metformin (1100 ± 70 ng/L) in effluent, and mefenamic acid (630 ± 3 ng/L), captopril (560 ± 11 ng/L), and amitriptyline (510 ± 1 ng/L) in influents. For radiopharmaceuticals, influent activity exceeded that in effluents, with Ga-67 being the most prominent (influent, 0.28 ± 0.07 Bq/L; effluent, 0.22 ± 0.02 Bq/L). Sludge samples showed higher radioactivity levels than wastewater samples, primarily from I-131 at 5 ± 2 Bq/kg. The data obtained from this study will enable the assessment of potential hazards posed by pharmaceutical mixtures in the receiving environment in a hitherto less studied region.

**Graphical abstract:**

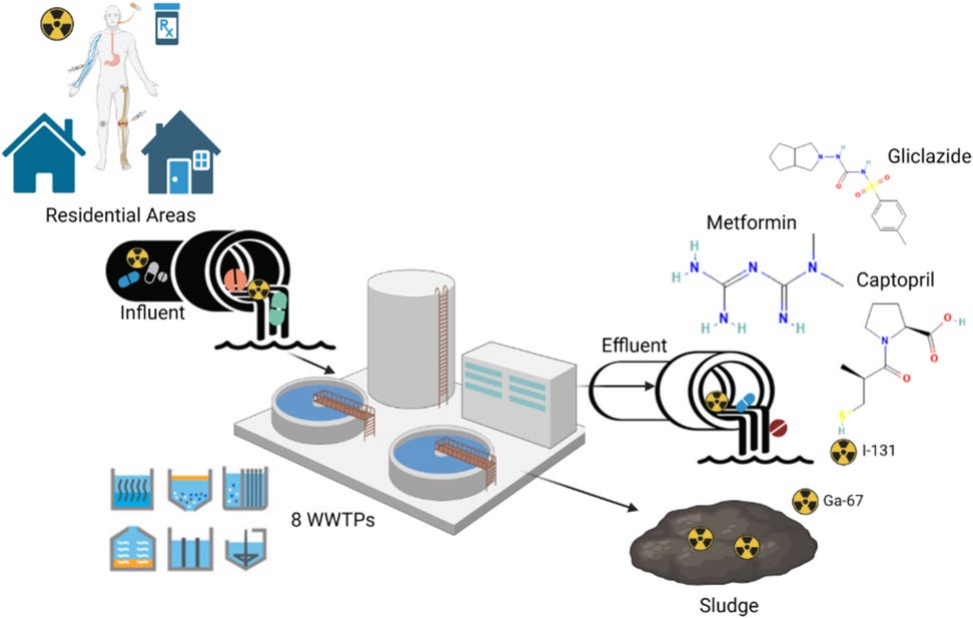

**Supplementary Information:**

The online version contains supplementary material available at 10.1007/s11356-025-36287-6.

## Introduction

Water scarcity is a pressing challenge in the Middle East and North Africa (MENA) region, where most of the world’s water-scarce countries are situated (Mahmoudi et al. 2023). Recycling sewage and wastewater is essential for tackling water scarcity (Chand et al. 2022). According to (UNICEF 2023), safe sanitation coverage in the MENA region had grown from 56 to 64% between 2015 and 2022. This coverage indicates the growth of wastewater treatment in the region. Depending on the effectiveness of the wastewater treatment plants (WWTPs), an affordable WWTP might produce water treated not to the potable water standard. However, it can still meet the safety requirements for various industries and industrial processes. In addition, there has been a rise in the utilisation of reclaimed water in the MENA region, with an average of 37% of treated wastewater being used for irrigation (Zekri & Al-Maamari 2020). For instance, Jordan and Libya have achieved impressive rates of 90% and 100%, respectively.

Douziech et al. (2018) found that chemical-specific processes, such as deconjugation and electrochemical interactions with sludge, play a crucial role in determining removal efficiencies (REs). These findings underscore the importance of evaluating individual chemicals, such as pharmaceuticals, to understand treatment performance better and improve predictive models for removing specific contaminants. The stability and biodegradability of compounds are often evaluated using pseudo-first-order kinetic models. Compounds with low values (< 0.01) are persistent (< 20% removed), those with moderate values (0.1–10) degrade moderately (20–90% removed), and those with high values (> 10) are easily biodegraded (> 90% removed) (Tran et al. 2018). Biodegradation is a primary mechanism for removing contaminants during biological treatment (Tran et al. 2018). However, many pharmaceuticals’ chemical and metabolic stability limits their removal (Finan et al. 2023; Perez-Lucas & Navarro 2024). For example, Clara et al. (2005) found that substances like carbamazepine are not significantly removed by WWTPs, with effluent concentrations similar to influent levels.

Globally, pharmaceuticals and other emerging contaminants are increasingly detected in influent, effluent, and water bodies, posing a significant environmental concern (Shi et al. 2018) as approximately 10% of pharmaceutical residues are expected to endure post-wastewater treatment (Abramova et al. 2020). As a result, a potential hindrance to expanding the use of treated wastewater for irrigation is the risk posed by emerging pharmaceuticals, as their detrimental effects spreading into waterways and the environment can be devastating. This issue has evolved in tandem with the high demand for pharmaceuticals, where the increased use of medicines worldwide leads to the likelihood of pharmaceuticals being released into wastewater through human excretions and waste.

Pharmaceutical substances like antibiotics, pain relievers, non-steroidal anti-inflammatory drugs, and antipsychotics can enter aquatic ecosystems (Zhang et al. 2020) and are not easily degradable (Kummerer 2009). Many of these pharmaceuticals are considered high-priority contaminants due to their potential environmental and human health risks. Their release into the environment can lead to ecological and human health issues as they accumulate in the surroundings. Therefore, it becomes crucial to detect and monitor the presence of pharmaceuticals in various fields, including clinical settings, human and animal food industries, and environmental toxicity and risk assessments.

Treatment processes at wastewater treatment plants (WWTPs) play an essential role in preventing the entry and establishment of emerging contaminants and diseases, which could dramatically affect the nation’s economy, environment, and way of life. Various WWTP technologies have been implemented today, each with distinct capabilities and advantages. A common type is the residential WWTP, which receives wastewater mainly from inhabited areas. These WWTPs are designed to treat wastewater and manage stormwater runoff, safeguarding freshwater resources and the environment. They employ various techniques to treat water before it can be reused or released into the environment. Emerging contaminants in the form of pharmaceuticals present a significant challenge for traditional WWTPs, which are not specifically designed for this purpose, resulting in partial removal (Miserli et al. 2022) depending on the chemical and physical properties of the pharmaceuticals and the treatment processes used.

Diverse techniques have been used to mitigate the presence of pharmaceuticals in wastewater. However, achieving complete elimination of pharmaceutical mixtures from reclaimed wastewater might be challenging, as conventional WWTPs are considered inefficient in dealing with the complexity of pharmaceutical compounds (Ba et al. 2014). Moreover, the evaluation of pharmaceutical risk is accompanied by several challenges that may impact the reliability of the findings. These challenges are associated with the physicochemical characteristics of pharmaceuticals, encompassing factors such as low water solubility, volatility, quick degradability, ionizability, absorptivity, and hydrophobicity, as well as the formation of complexes and presence within complex mixtures (Ye et al. 2022). Some research on pharmaceuticals in WWTPs has been conducted in various nations in the MENA region. Table 1 lists the pharmaceuticals detected in wastewater samples from MENA. Nevertheless, the knowledge regarding emerging pharmaceuticals in the MENA region’s wastewater is still limited.

**Table 1:** Occurrence of pharmaceuticals in the influents and effluents of WWTPs in different geographical countries in the MENA regions

No	Country	Treatment process	Treatment plant capacity	Region (rural/urban)	Matrix	Range of LOD and LOQ	Detected compound (s)	Concentration in untreated/influent	Concentration in treated/effluent	References
1	Kuwait	−	−	Urban	HWW discharge (untreated)	−	Metronidazole Sulfamethoxazole Trimethoprim Paracetamol	400–45,800 ng/L 30–710 ng/L 10–110 ng/L 50,000–179,000 ng/L	−	(Mydlarczyk et al. 2022)
2	UAE	−	−	Urban	Effluent	LOD: 0.1 and 2 ng/L LOQ: 0.3–5 ng/L for all analytes	Sulfapyridine Sulfamethazine Sulfadiazine Sulfamethoxazole Ciprofloxacin Ofloxacin Erythromycin Paracetamol Metoprolol Risperidone	90–420 ng/L 10–40 ng/L 550–890 ng/L 100–230 ng/L 700–1000 ng/L 680–1000 ng/L 620–950 ng/L 144,000–147,000 ng/L 80–110 ng/L 190–300 ng/L	90–110 ng/L 7–15 ng/L 270–600 ng/L 70–80 ng/L 380–710 ng/L 350–680 ng/L 380–710 ng/L 3900–6600 ng/L 50–80 ng/L 10–15 ng/L	(Semerjian et al. 2018)
3	Iran	WWTP1, WWTP2, WWTP3: extended aeration WWTP4: A2O processes	Populations WWTP1: 100,000, WWTP2: 85,000, WWTP3: 2000, WWTP4: 30,000	Urban	Influents, effluents, and HWW	LOQs for IBP, NPX, DIC, and IDM were 20, 30, 20, and 20 ng/L	Ibuprofen Naproxen Diclofenac Indomethacin	Municipal WWTP: 230–1100 ng/L 90–430 ng/L 40–230 ng/L 40–110 ng/L HWW.: 140–290 ng/L 80–90 ng/L 30–80 ng/L 30–40 ng/L	Municipal WWTP: 30–50 ng/L 30–50 ng/L 20–30 ng/L 30–60 ng/L	(Eslami et al. 2015)
4	Iran	WWTP A: anaerobic, anoxic, oxic WWTP B: conventional activated sludge (CAS) and tricking filter beds	WWTP A: 100,000 residents; capacity 24,000 m^3^/day WWTP B: 2.1 million people; capacity of 112,300 m^3^/day	Urban	Influent and effluent	1–3 ng\L	Ciprofloxacin Amoxicillin Penicillin Cefixime Cephalexin Azithromycin Erythromycin	WWTP A: 550–800 ng/L 110–590 ng/L 13–36 ng/L < LOQ–320 ng/L 120–460 ng/L < LOQ–40 ng/L 60–160 ng/L WWTP B: 400–7100 ng/L 200–330 ng/L 34–66 ng/L 270–780 ng/L 520–980 ng/L − 20–93 ng/L	WWTP A: 130–250 ng/L 24–95 ng/L < LOQ–17 ng/L 50–120 ng/L < LOQ–30 ng/L 9–15 ng/L 30–51 ng/L WWTP B: 35–210 ng/L 60–100 ng/L 8–31 ng/L 280–420 ng/L − 160–230 ng/L 140–190 ng/L	(Mirzaei et al. 2018)
5	Algiers	Mechanical and biological treatments	WWTP-1: 80,000 m^3^/day for 400,000 inhabitants WWTP-2: 50,400 m^3^/day for 250,000 inhabitants	Urban	Influent and effluent	LOD, LOQ: IBU: 0.5, 2 ng\L; NAP: 16, 55 ng\L; KET: 0.5, 2 ng\L; DIC: 3, 10 ng\L	Ibuprofen Naproxen Ketoprofen Diclofenac	1600–8600 ng/L 1200–9600 ng/L ND–570 ng/L 990–2300 ng/L	340–430 ng/L ND–330 ng/L ND–1030 ng/L 1600–2700 ng/L	(Kermia et al. 2016)
6	Tunisia	Primary (settling and flotation) and secondary CAS	Populations: 500,000 inhabitants	Urban	Effluent	LOD < 0.5 µg/L	Carbamazepine Naproxen Ibuprofen	−	ND–130 µg/L ND–36 µg/L ND–43 µg/L	(Khazri et al. 2019)
7	Tunisia	Pretreatment, primary settling, and CAS	Cover 4 metropolitan area	Urban	Influent and effluent	< 0.5 ng m/L	Chloramphenicol Thiamphenicol Florfenicol Paromomycin Dihydrostreptomycin Kanamycin B Aparamycin Streptomycin Amikacin Sisomycin Neomycin Gentamycin c1a Gentamycin c1 Gentamycin c2	ND–3 µg/L ND–1 µg/L 1–3 µg/L ND–4 µg/L ND–1 µg/L 0.5–8 µg/L ND–1 µg/L ND–3 µg/L ND–2 µg/L ND–7 µg/L 2–16 µg/L ND–2 µg/L ND–0.8 µg/L ND–1 µg/L	ND–1 µg/L ND 0.1–0.8 µg/L ND–1 µg/L ND–0.4 µg/L ND–5 µg/L ND–0.5 µg/L ND–1 µg/L ND–2 µg/L ND–4 µg/L 0.4–11 µg/L ND–0.6 µg/L ND–0.4 µg/L ND–0.3 µg/L	(Tahrani et al. 2016)
8	Qatar	WWTP: conventional aeration and CAS; tertiary treatment via sand filter NWWTP: anaerobic selector, anoxic selector, and extended aeration with activated sludge recirculation; tertiary treatment via sand filter and ultrafiltration	OWWTP: flow rate: 175,500 m^3^/day NWWTP: flow rate: 54,000 m^3^/day	Urban	Influents, effluents, and HWW effluent	LOD: 10 ng/L	Clavulanic acid Metronidazole Amoxicillin Ciprofloxacin Tetracycline Penicillin Erythromycin	WWTPs: 4300–100,000 ng/L < LOD–3100 ng/L 70–280 ng/L 230–2500 ng/L 200–320 ng/L 8–690 ng/L < LOD–2040 ng/L HWW: < LOD–41,200 ng/L 970–5500 ng/L < LOD–800 ng/L 330–2000 ng/L < LOD–200 ng/L < LOD–120 ng/L < LOD–5200 ng/L	WWTPs: 3400–44,700 ng/L < LOD–330 ng/L < LOD–80 ng/L 160–1700 ng/L < LOD–260 ng/L < LOD–420 ng/L < LOD–170 ng/L	(Al-Maadheed et al. 2019)
9	Jordan	−	Populations: 1.9 M inhabitants	Urban	Treated WW (TWW), surface water (SW), and groundwater (GW)	LOD (pharmaceuticals): 20 ng/L in GW and SW, 50 ng/L in TWW. LOD (ICM): 10 ng/L in GW and SW. 50 ng/L in TWW	Bezafibrate Carbamazepine Clofibric acid Diazepam Diclofenac Fenofibrate Fenofibric acid Gemfibrozil Ibuprofen Ketoprofen Naproxen	−	Max conc. (ng/L) in TWW; SW; GW: 480; 390; − 17,000; 2100; 500 150; 33; − 720; 13; − 430; 160; − 260; − ; 74 160; − ; − 4800; 2100; − 750; 1400; 59 64; − ; − 240; 550; −	(Zemann et al. 2014)
10	Palestine	Two greywater treatment systems: 1 and 2: constructed wetlands greywater reuse system 3 and 4: up-flow gravel filtration greywater reuse systems	−	Rural	Household greywater and treated greywater effluent	LOD: 0.5 µg/L	Azithromycin Ciprofloxacin Erythromycin Linezolid Oxacillin Oxolinic acid Penicillin G Pipemidic acid Sulfamethoxazole triclocarban Tetracycline Vancomycin Caffeine	0.3–78 ng/L 0.4–198 ng/L 0.2–13 ng/L 0.3–13 ng/L 0.9–410 ng/L 0.5–150 ng/L 0.7–160 ng/L 0.4–320 ng/L 0.5–80 ng/L 0.7–1600 ng/L 0.5–890 ng/L 0.3–13 ng/L 2800–410,000 ng/L	0.3–25 ng/L 0.4–87 ng/L 0.2–20 ng/L 0.3–2 ng/L 0.9–270 ng/L 0.5–300 ng/L G 0.7–63 ng/L 0.4–62 ng/L 0.5–18 ng/L 0.7–150 ng/L 0.5–2000 ng/L < LOD 40–190,000 ng/L	(Craddock et al. 2020)
11	Jordan	As-Samra WWTP CAS/extended aeration system Wadi Al-Seer WWTP aerated lagoon treatment method	As-Samra WWTP capacity 365,000 m^3^/day Wadi Al-Seer WWTP capacity 4000 m^3^/day	Urban	Influent	LOD: 5 ng/L	1,7-Dimethylxanthine Acetaminophen Amphetamine Caffeine Carbamazepine Cimetidine Cotinine Diphenhydramine MDA MDMA Methamphetamine Morphine Phenazone Sulfachloropyridazine Sulfamethazine Sulfamethoxazole Thiabendazole Trimethoprim	5900–7500 ng/L 140–28,700 ng/L < LOD 30,000–182,500 ng/L 550–1500 ng/L < LOD 60–4700 ng/L < LOD < LOD < LOD–20 ng/L < LOD 40–90 ng/L < LOD–40 ng/L < LOD < LOD–20 ng/L 250–350 ng/L < LOD–10 ng/L 20–130 ng/L	−	(Tahrani et al. 2016)
12	Tunisia	Six WWTPs: biologic – CAS and secondary biological treatment One WWTP: biologic – aerated lagoon	5000–78,000 m^3^/day; for 30,000–600,000 population	Urban	Influent and effluent	Illicit drugs: LOD: 1–25 ng/L, LOQ: 3–83 ng/L	Paracetamol Caffeine Propranolol Atenolol Carbamazepine Erythromycin Clarithromycin Ofloxacin Ciprofloxacin Sulfamethoxazole Trimethoprim Amphetamine Methylenedioxymeth-amphetamine (MDMA) Methamphetamine Cocaine Benzoylecgonine	< LOD–1200 ng/L 2400–59,400 ng/L < LOQ 60–2200 ng/L < LOQ–270 ng/L < LOD–1200 ng/L < LOD–120 ng/L 140–870 ng/L < LOQ–210 ng/L < LOD–200 ng/L < LOD < LOQ–80 ng/L < LOQ–150 ng/L < LOD–120 ng/L < LOQ/ < LOD–450 ng/L < LOQ–100 ng/L	< LOD–410 ng/L 1060–34,600 ng/L < LOQ 420–1200 ng/L 180–340 ng/L 320–1200 ng/L < LOD 190–650 ng/L < LOQ 35–270 ng/L < LOQ/ < LOD < LOQ < LOQ/ < LOD < LOD < LOQ/ < LOD–230 ng/L < LOQ	(Moslah et al. 2018)
13	Tunisia	Sud Meliane: **p**retreatment and a secondary biological treatment using oxidation ditches SE4 and SE3: treatment process is not specified WWTP Korba: secondary treatment by oxidation ditches process and tertiary treatment through maturation ponds	Sud Meliane: 40,000 m^3^/day SE4: 9600 m^3^/day SE3: 3500 m^3^/day WWTP Korba: 7500 m^3^/day	Urban	Influent and effluent	LOQ: 10 ng/L–200 ng/L LOD: 3 ng/L–67 ng/L	Diclofenac Furosemide Ketoprofen Naproxen Atenolol Fenofibric acid Carbamazepine Ibuprofen 1-OH-ibuprofene 2-OH-ibuprofene	32–700 ng/L 32–700 ng/L 32–700 ng/L 32–700 ng/L 1800 ng/L 1250 ng/L 32–700 ng/L 1600 ng/L 110 ng/L 730 ng/L	> 1000 ng/L > 1000 ng/L > 1000 ng/L > 1000 ng/L > 1000 ng/L > 1000 ng/L 190–1300 ng/L > 1000 ng/L > 1000 ng/L > 1000 ng/L	(Fries et al. 2016)
14	Saudi Arabia	Tertiary treatment	300,000 m^3^/day	Urban	Influent and effluent	LOD: 0.2–6 µg/L	Metformin Atenolol Cephalexin Paracetamol Norfluoxetine	4–31 ng/L < MDL–4 ng/L < MDL–3 ng/L 4–100 ng/L < MDL–10 ng/L	< LOD–5 ng/L < LOD–2 ng/L < LOD–3 ng/L < LOD–90 ng/L < LOD–10 ng/L	(Shraim et al. 2017)
15	Saudi Arabia	WWTP1-1: primary clarification; biological treatment WWTP1-2: secondary clarification; multi-media filtration; chlorination WWTP2-1: screening; membrane bioreactor (MBR) WWTP2-2: (nitrifying/denitrifying); chlorination WWTP 3: primary clarification; biological nutrient removal (nitrifying/denitrifying) including bio-P; U.V. disinfection WWTP 4: primary clarification; biological nutrient removal (nitrifying/denitrifying); chlorination	WWTP 1 flow rate: 80,000 m^3^/day WWTP 2 flow rate: < 4000 m^3^/day WWTP 3 flow rate: 74,000 m^3^/day WWTP 4 capacity: 19,700 m^3^/day	Urban	Effluent	LOD in MilliQ: 0.2–2 ng/L LOQ in MilliQ: 0.3–30 ng/L LOQ: 6–300 ng/L	Caffeine Carbamazepine Dilantin Primidone Sulfamethoxazole Trimethoprim Diclofenac Ibuprofen Naproxen Amitriptyline Fluoxetine Atenolol Diphenhydramine Gemfibrozil Triclocarban Triclosan Methylparaben Propylparaben	−	< 60–16,500 ng/L 57–1200 ng/L < 20–440 ng/L < 3–650 ng/L 150–730 ng/L - < 10–790 ng/L 30–1260 ng/L < 10–930 ng/L < 6–310 ng/L 12–370 ng/L 10–300 ng/L 15–2550 ng/L 50–760 ng/L 6–430 ng/L 40–500 ng/L < 250–2370 ng/L < 30–920 ng/L < 20–590 ng/L	(Alidina et al. 2014)
16	Saudi Arabia	CAS process with aerobic process	HWWTP1: 330,000 m^3^/year HWWTP2: 227,000 m^3^/year	Urban	Influents and effluents	LOQ: 0.5–3 ng/L	Paracetamol Carbamazepine Atenolol Lidocaine Ciprofloxacin Clarithromycin Sulfamethoxazole NACS Caffeine	12,300–12,400 ng/L 73–150 ng/L 330–730 ng/L 130–160 ng/L 2200–5600 ng/L 38–83 ng/L 30–130 ng/L 510–1200 ng/L 27,500–74,800 ng/L	70–160 ng/L ND–41 ng/L 50–55 ng/L < LOD–110 ng/L ND ND–20 ng/L ND ND–60 ng/L ND	(Al Qarni et al. 2016)
17	Jordan	−	−	Rural and urban	Influent, effluent, and HWW effluent	LOD: 0.6–3 µg/L LOQ: 0.9–6 µg/L	Methotrexate Caffeine Diclofenac Glimepiride Ibuprofen	WWTPs: 100–450 µg/L 420–1080 µg/L 7–65 µg/L 4–100 µg/L 5–62 µg/L HWW: 180–840 µg/L 45–400 µg/L 3–7 µg/L 3–10 µg/L ≤ 26 µg/L	WWTPs: 95–330 µg/L 150–570 µg/L ≤ 44 µg/L ≤ 32 µg/L ≤ 49 µg/L	(Alahmad & Alawi 2010)
18	Jordan	Primary skimming and screening and secondary waste stabilisation ponds	−	Urban	Influent and effluent	Instrumental LOD: 0.05 and 20 pg Spiked influent and effluent LOQ: 500 and 200 μg/L	Carbamazepine Sulfamethoxazole Erythromycin Ibuprofen Naproxen Diclofenac Ketoprofen	700–3600 μg/L ND–900 μg/L 500–1100 μg/L ND–5700 μg/L 700–5200 μg/L 800–3300 μg/L ND	400–1300 μg/L ND–300 μg/L ND–500 μg/L ND–2200 μg/L ND–1300 μg/L ND–1100 μg/L ND	(Al-Tarawneh et al. 2015)
19	Iran	−	−	Urban	Influent, affluent, and HWW effluent	−	17β-estradiol	WWTPS: 42–98 ng/L HWW: 93–83 ng/L	WWTPs: 9–23 ng/L	(Hassani et al. 2016)

*WWTP*, wastewater treatment plant; *HWWTP*, hospital wastewater treatment plant; *WW*, wastewater; *HWW*, hospital wastewater; *ND*, not detected; *LOD*, limit of detection; *LOQ*, limit of quantification.

Moreover, the utilisation of nuclear medicine procedures has markedly risen in the last decade, with considerable variability observed among countries (Yu et al. 2019). Consequently, the usage of radiopharmaceuticals is anticipated to grow. Radiopharmaceuticals are a group of pharmaceutical products widely used for oncological diseases (therapy or diagnostics). Generally, the three building blocks of radiopharmaceuticals are the radioisotope, a targeting biomolecule (bio-vector), and a bifunctional agent (conjugation, pharmacokinetics) (Sarko et al. 2012). The eliminated radionuclides can reach WWTPs after discharge directly from the diagnosis dosages or indirectly after administering treatment regimens. Hay et al. (2011) found I-131 and Tc-99 m in the treated wastewater effluent from the Klamath Falls WWTP.

Mulas et al. (2019) and Todorov et al. (2020) suggested that binding radiopharmaceuticals with suspended organic particles in sewage systems and WWTP treatment processes maintained radioactivity beyond their known radioactive decay. Radiopharmaceuticals can be assimilated into the soil matrix, exhibiting reduced movement rates within the unsaturated zone (Walther & Gupta 2015). Consequently, a significant portion of the initially deposited radioactive content might persist within the root-encompassing region for extended periods, presenting a considerable likelihood of entering the food chain. The authors held the view that the interaction patterns of radionuclides within soil systems are contingent upon both their elemental attributes and the physicochemical traits of the soil. This intricate interplay becomes more elaborate due to the concurrent operation of numerous processes influenced by prevailing environmental conditions. These radionuclides affect people and ecosystems regardless of exposure (Hossain 2020). Numerous studies in radioecology focus on the emission of artificial radionuclides into the surroundings. Still, only a limited number of studies investigated medical radiopharmaceuticals in water, sludge, and the surrounding environment. Table 2 compiles data on radiopharmaceuticals studied in the MENA region and other countries beyond it.

**Table 2:** Occurrence of radiopharmaceuticals in the influents and effluents of WWTPs in different geographical regions

No	Country	treatment process	Treatment plant capacity	Region (rural/urban)	Matrix	Detected compound (s)	Untreated/influent	Treated/effluent	Other samples	References
1	Korea	Two dehydration systems, 2 inflow systems, and 2 discharge outlets	0.3 million tons WW/day	Urban	WW influent, effluent, and sludge	I-131 99mTc	0.3–4 Bq/L	0.4–2 Bq/L	Digested sludge; dewatered sludge 200–230 Bq/kg; 1960–2410 Bq/kg < MDA; < MDA–26 Bq/kg	(Chang et al. 2011)
2	Kuwait	−	−	Urban	HWW discharge (untreated)	I-131 99mTc	14–27 Bq/L 0.14–14,150 Bq/L	−	−	(Mydlarczyk et al. 2022)
3	Oman	Two delay tanks	40 kL	Urban	WW	I-131	Max. released in 4 years: 5 MBq /m^3^	−	−	(Ravichandran et al. 2011)
4	Oman	Holding tanks for irrigation	Serves 10,300 inhabitants	Urban	Irrigation water and sludge	131I	−	2–20 Bq/L	12–460 Bq/kg	(Bererhi & ConsTable 1999)
5	Indonesia	Building 1: decay tank Building 2**:** advanced waste treatment system connected to the HWWTP, discharging into local rivers	−	Urban	WW and air	I-131	Decay tanks and temporary collection channels: 96 × 10^+6^ ± 4 × 10^+6^ Bq/m^3^ After mixing with liquid waste from other units: 472,680 ± 22,160 Bq/m^3^	HWWTP outlet: 37,700 ± 2040 Bq/m^3^	Air in the open space for the 2 nuclear medicine buildings: 1 ± 0.27 Bq/m^3^	(Puspita et al. 2023)
6	USA	Conventional activated sludge (CAS)	14.9 m^3^/s for nearly 600,000 residents	Urban	Effluents and sediment	I-131	−	Bulk effluent: 250 ± 50 mBq/L	Surficial sediment samples from 6 sites: < MDL–6 ± 1 mBq/g	(Smith et al. 2008)
7	Barcelona Metropolitan Area, Spain	CAS, membrane bioreactor (MBR), and integrated fixed-film activated sludge system (IFAS)	TP-1 and TP-2: > 200,000 m^3^/day for 2 million inhabitants, TP-3, TP-4, and TP-5: 40,000–80,000 m^3^/day TP-6 and TP-7: < 1000 m^3^/day	Urban	Influent, effluents, and sewage sludges	131I 99mTc 111In 67 Ga 123I	0.32 ± 0.16–4 ± 0.29 Bq/L 7 ± 1–50 ± 3 Bq/L 0.26 ± 0.16–0.28 ± 0.13 Bq/L < MDA < MDA –0.64 ± 0.44 Bq/L	0.23 ± 0.11–3 ± 0.23 Bq/L 5 ± 0.65–7 ± 1 Bq/L 0.17 ± 0.093–0.26 ± 0.059 Bq/L < MDA < MDA	41 ± 6–1070 ± 32 Bq/kg 70 ± 5–6420 ± 280 Bq/kg 5 ± 1–33 ± 3 Bq/kg 6 ± 4–83 ± 18 Bq/kg 6 ± 4–56 ± 6 Bq/kg	(Mulas et al. 2019)
8	Bremen, Germany	Mechanical stage, producing primary sludge, biological stage	50 million m^3^/year for 500,000 inhabitants	Urban	Influent, effluent, primary sludge, sewage sludge, and river sediment	7Be 99mTc 123I 131I 137Cs 153Sm	− 1–19 Bq/L − 0.17–0.86 Bq/L − −	− 0.067–4 Bq/L − 0.037–0.98 Bq/L − −	Primary sludge; sewage sludge; river sediment 28–320; 4–560; 18–230 140–6040; 0.2–250; (-) 3–34; (-); (-) 10–570; 0.1–200; 0.1–110 0.98–4; 0.1–32; 3–22 7–165; (-); (-)	(Fischer et al. 2009)
9	Stony Brook, NY, USA	Tertiary treatment via an oxidation ditch with CAS, mixed liquor returns, and sodium hypochlorite (disinfectant)	Serves 20,000 inhabitants	Urban	Effluent and suspended solids > 0.7 μm	131I	−	Unfiltered effluent: 2 ± 0.3 to 220 ± 1 Bq/L Filtered effluent: 2 ± 0.3 to 230 ± 2 Bq/L Suspended solids: 61 ± 12 to 2800 ± 32 Bq/g	−	(Rose et al. 2012)
10	North-western Sicilian coast, Italy	Primary treatments and secondary treatments (CAS)	WWTP-1: 153,600 m^3^/day, WWTP-2: 19,704 m^3^/day, WWTP-3: 44,448 m^3^/day	Urban	Influent, effluent, and dehydrated sludge	131I Note: 137Cs, 111In, 99Mo-99mTc and 67 Ga < MDA	5 Bq/L	0.90 Bq/L	Dehydrated sludge: 12 Bq/kg	(Cosenza et al. 2015)
11	Catalonia, Spain	CAS with anaerobic digestion	Reus WWTP: 25,000 m^3^/day for 195,833 inhabitants Tarragona WWTP: 35,000 m^3^/day for 175,000 inhabitants	Urban	Dehydrated sludge	131I 99mTc 67 Ga 111In	−	−	**The Reus WWTP:** 180–210 Bq/kg − 20 Bq/kg − **Tarragona WWTP:** 13–13 Bq/kg − 11 Bq/kg 3 Bq/kg	(Martinez et al. 2018)

*WWTP*, wastewater treatment plant; *HWWTP*, hospital wastewater treatment plant; *WW*, wastewater; *HWW*, hospital wastewater; *MDA*, minimum detectable activity.

Hence, the primary aim of this research was to evaluate the presence and concentration of pharmaceuticals, including radiopharmaceuticals, in influents and effluents of WWTPs. The country studied lacks comprehensive information on pharmaceutical detection in WWTPs and other environmental water sources. This critical information aids in understanding the environmental factors contributing to pharmaceutical-related risks, such as the proliferation of antimicrobial resistance, and supports the development of effective, evidence-based strategies to mitigate these risks.

## Materials and methods

**Fig. 1:**
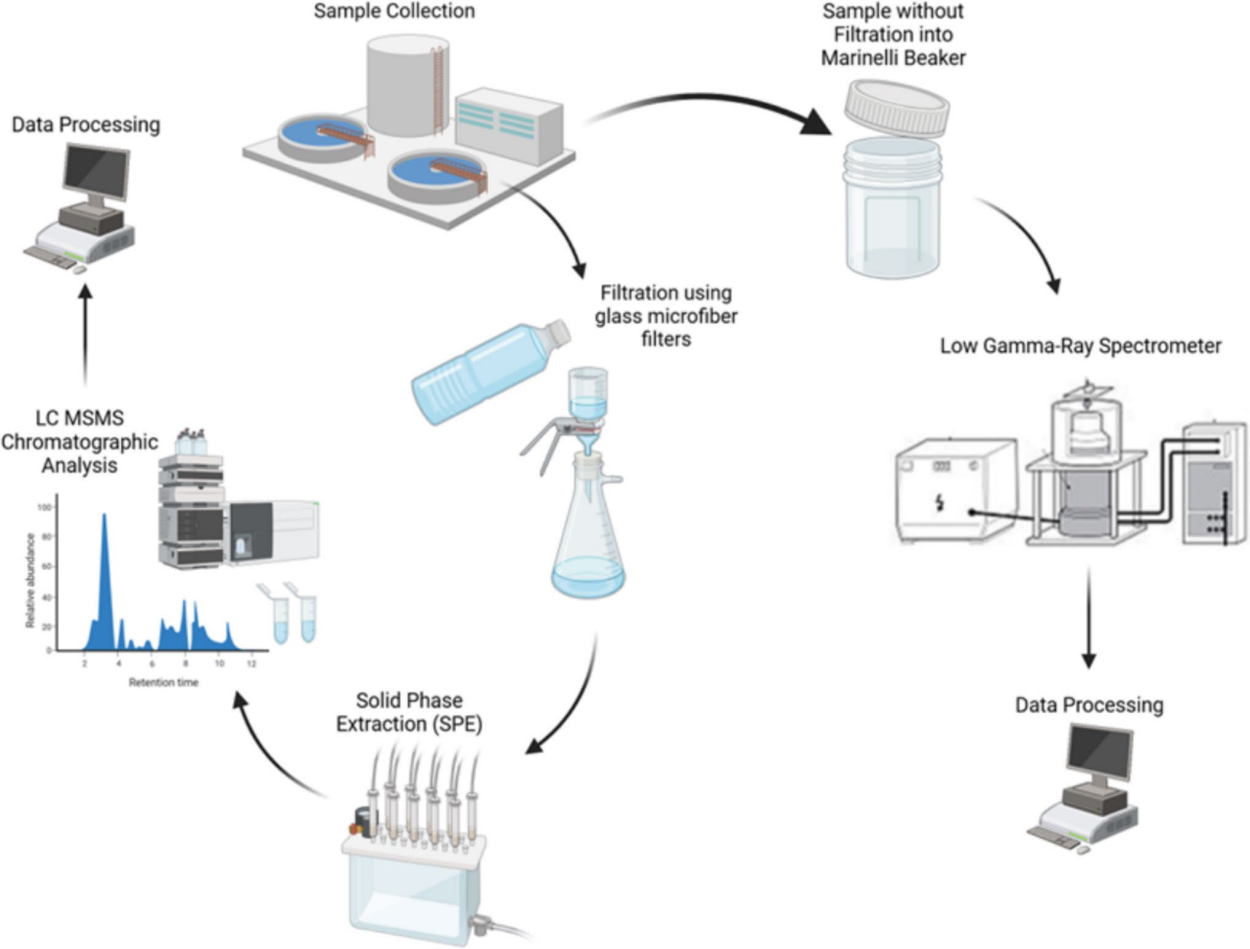
Overall samples analysis for determination of pharmaceuticals and radiopharmaceuticals in the selected samples

A general overview of the methodology for determining the pharmaceuticals and radiopharmaceuticals in the selected samples is illustrated in Fig. 1.

### Site selection

WWTPs worldwide follow a standard process consisting of primary, secondary, tertiary treatment, and disinfection stages. The selected sites for this study have mechanical processes designed to remove wastewater constituents, thereby producing treated effluent in compliance with regulations and permitting it to be used safely for other purposes. There were 59 urban and rural WWTPs in the country when this study was conducted, with eight plants in the capital and 51 plants distributed across different regions.

The samples collected for this study were taken from plants utilising different wastewater treatment technologies, including conventional activated sludge (CAS), a membrane bioreactor (MBR), and a sequential batch reactor (SBR) with an ultrafiltration system (U.F.). Table S1 provides detailed information about the WWTPs from which samples were obtained. Each treatment technology involves a series of processes to eliminate contaminants and reduce pollutants in the wastewater stream.

### Sample selection

In this study, the samples collected from domestic (municipal) WWTPs were primarily water (99%), with relatively modest concentrations of suspended and dissolved organic and inorganic solids. Information about the major physical and chemical parameters of influents and effluents in the studied WWTPs is summarised in Table S2.

### Sample collection, extraction, and analysis

Samples were collected during winter, December 2021–February 2022, before treatment (influent) and after treatment (effluent), and sludge samples from eight WWTPs according to standard sampling procedures. Influent and effluent samples were collected by grab sampling after rinsing the container (pre-cleaned and acid-treated dry amber glass bottles) several times with the water sample. The container was filled with no headspace and closed. In addition, a composite sampling procedure was used to collect samples. An equal portion of the samples collected was combined using the procedure above to obtain samples in the field. If using an auto-sampler with multiple bottle configurations, the sample bottles were taken out from the auto-sampler, mixed well, and a cautiously equal proportion of samples combined. The combined sample was poured into the sample container. The sample was mixed well for one bottle configuration, poured into the sample container and closed. To detect radiopharmaceuticals, wastewater influents, effluents, and sludge (dried treated) samples were filled directly into Marinelli cylindrical beakers (the container used to measure radioactivity). Table S3 includes information about the samples collected from different locations in this study.

The analytical methods described by Roberts et al. (2016) were applied in this study unless stated otherwise. Analytical standards were supplied by Sigma Aldrich (MO, USA). High-purity solvents, including methanol, acetone and dichloromethane, were provided by Fisher Scientific (Leicestershire, UK). Stock standard solutions of analytes were prepared in methanol in amber glass bottles that were cleaned with solvents and heat-treated. These solutions were diluted in amber glass vials to create serial low-concentration working solutions and calibration sets. All standard mixtures were stored at − 20 °C.

During field collection and transportation, all samples were stored at 4 °C or lower temperatures. Water samples were immediately acidified with H_2_SO_4_ upon collection to prevent microbial activity. Upon arrival at the laboratory (within 72 h), samples were promptly extracted. Sample preparation for chemical analysis was performed by prefiltration of 500 mL wastewater samples using 1.2 um GA-55 Advantec® glass fibre filter paper (Toyo Rochi Kaisha Ltd.; Tokyo, Japan) before their enrichment via preconditioned 6 mL (1 g) SupelcleanTM ENVI-18 SPE cartridges (SUPELCO Analytical; P.A., USA). SPE cartridges were conditioned by passing 5 mL of methanol twice, then rinsing with 5 mL of Milli-Q water twice. Filtration was performed using a Preppy™ vacuum manifold (SUPELCO Analytical; P.A., USA). The SPE cartridge was eluted thrice with 3 mL of methanol each, followed by three elution steps with 3 mL of dichloromethane each. Subsequently, the solvents were entirely evaporated under N2 gas blow, and then the sample was reconstituted with 500 µL of methanol in an amber glass vial. Then, the quantification of the pharmaceutical residues was carried out using LC-MSMS Thermo Finnigan TSQ Quantum Discovery Max (Thermo Electron Corporation; F.L., USA) coupled with an Agilent 7000A triple quadrupole mass spectrometer (MSMS).

To detect radiopharmaceuticals, fifteen fresh (non-filtered) wastewater, recycled water, and dehydrated sludge samples from a WWTP were filled into 1 L Marinelli cylindrical beakers. The radioactivity was using low gamma-ray spectrometry equipped with a gamma-ray spectrum analyser with a high purity germanium (HPGe) detector from Ortec® (AMETEK; T.N., USA) connected by computerised GammaVision® software and multichannel analyser (MCA) Emulators for Microsoft Windows®. The detector is shielded with low background lead. The spectrometer was calibrated using a mixed gamma nuclide standardised solution EZ-8501-EG-SD (Eckert & Ziegler Isotope Products; C.A., USA). Samples were processed within not more than 12 h from the collection and counted for 5 h with high-resolution gamma-ray spectrometers (HPGe detectors).

### Scope of analysis

This study included the analytical determination of 19 pharmaceuticals with different physicochemical properties from different classes. These included four antimicrobials: erythromycin (ERY), metronidazole (MNZ), ofloxacin (OFX), and trimethoprim (TMP); two antihyperglycemics: gliclazide (GLZ) and metformin (MTF); two antilipemic: atorvastatin (ATS) and simvastatin (SVS); two β-blockers: atenolol (ATN) and propranolol (PNL); two antihypertensive: captopril (CTP) and lisinopril (LSP); urological agent: sildenafil (SDF); antidepressant: amitriptyline (AMT); two antihistamines: chlorpheniramine maleate (CPM) and diphenhydramine (DPH); antispasmodic: hyoscine butyl bromide (HBB); analgesic/antipyretic: paracetamol (PCM); and a non-steroidal anti-inflammatory: mefenamic acid (MFA). Table S4 describes the physico-chemical characteristics of the targeted pharmaceuticals. The pharmaceuticals investigated in this study were chosen based on their occurrence, measured wastewater concentrations worldwide, and analytical feasibility.

### Performance of the method

Tandem MS analysis was undertaken using electrospray ionisation in positive ionisation modes, and the M.S. detector was operated in MRM mode (MRM listed in Table S5). The method’s performance was assessed by evaluating linearity, accuracy—expressed as the recovery percentage during extraction—and precision, defined by relative standard deviation. The results demonstrated good linearity, with determination coefficients exceeding 0.99, assessed across twelve matrix-matched calibration points within the 1–500 µg/L range for all compounds under investigation. A total of nineteen pharmaceutical reference standards were employed, with mixtures prepared at concentrations of 1, 3, 5, 10, 20, 30, 50, 70, 100, 200, 300, and 500 µg/L. Accuracy and precision were further evaluated by spiking wastewater samples prior to the filtration step at concentrations of 0.1 and 0.5 µg/L, with three replicates at each level. To evaluate recoveries, the concentrations measured following the entire SPE procedure were compared with the initial spiking concentrations, with reference standard calibration used for determination. Method recovery rates varied from 81% for atenolol to 102% for trimethoprim in influents and from 83% for atorvastatin to 107% for trimethoprim in effluents. The mean percentage recovery of the identified compounds in quality control samples and the reported recoveries obtained through a comparable methodology are presented in Table S6.

The limit of detection (LOD) and limit of quantification (LOQ) were determined for each analyte. The LOD was computed based on the standard deviation of the response (Sy) from each reference standard curve and the slope of the calibration curve (S) at levels approximating the LOD employing the formula LOD = 3.3(Sy/S). The determination of the standard deviation of the response was based on the standard deviation of the y-intercepts of regression lines. LOQ was generally found to be approximately three times greater than the LOD, calculated by the formula LOQ = 10(Sy/S). The values for LOD and LOQ are listed in Table S7, ranging from 6.6 to 42.4 ng/L for LOD and 20.0 to 128.4 ng/L for LOQ.

### Analysis of radiopharmaceuticals

The studied radiopharmaceuticals (technetium-99 m (Tc-99 m), 18fluorine-fluorodeoxyglucose (F-18), gallium-67 (Ga-67), and iodine-131 (I-131)) were chosen based on their availability and usage in the studied nation, occurrence in wastewater worldwide, and their analytical feasibility by application of gamma spectroscopy. Table S8 presents the physical-radioactive characteristics of the targeted radiopharmaceuticals. Sample weights were recorded for results quantification. The minimum detectable activity (MDA) of an HPGe‐detector was established based on factors including its energy resolution, crystal efficiency, peak/compton‐factor, background, measuring time, sample shape, self‐absorption, and emission probabilities of gamma radiation lines emitted by the radionuclide. Peak analysis was performed using Gamma Vision™ software. The obtained Gamma spectra were stripped of background counts and corrected according to their density.

## Results and discussion

### Pharmaceuticals levels in eight heterogeneous WWTPs

**Fig. 2:**
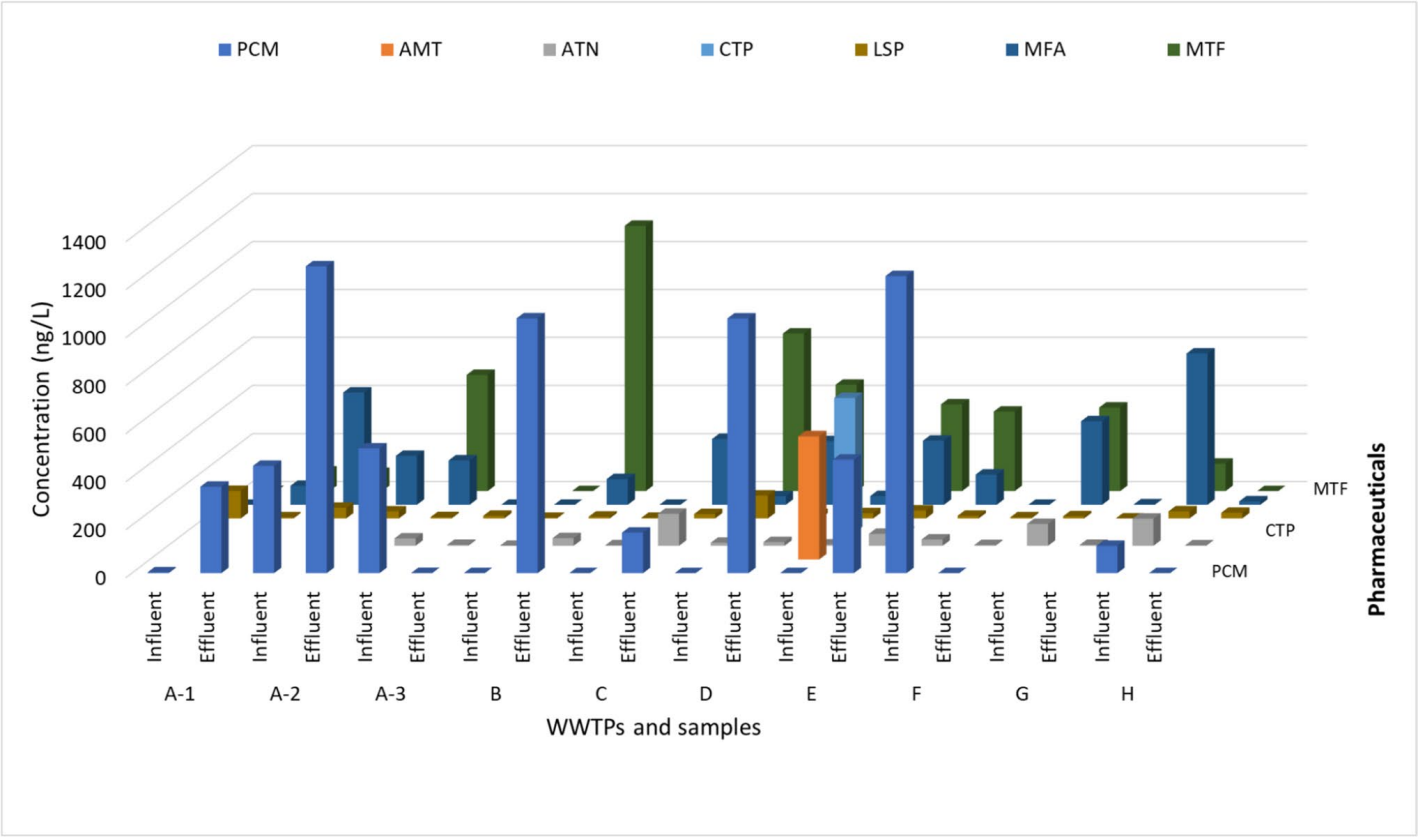
Overview of concentrations of pharmaceuticals detected in influent and effluent samples from selected eight urban and rural WWTPs

The analysis identified thirteen pharmaceuticals, including ERY, MNZ, GLZ, MTF, ATS, ATN, CTP, LSP, SDF, AMT, DPH, MFA, and PCM (refer to Fig. 2). Table 3 shows the maximum concentration, detection frequency percentage, and relative abundance percentage of the studied pharmaceuticals in the WWTPs. Among these compounds, ten were previously reported in various studies conducted in the MENA region (Table 1). Remarkably, the compounds GLZ, ATS, CTP, LSP, SDF, and MFA identified in this study were not previously reported in the MENA region. Among these, LSP and MFA, along with MTF, ATN, and PCM, were the most frequently detected pharmaceuticals in this study, with a detection frequency reaching 75 to 100%. Other compounds from the same or different categories were also detected with lower frequency values, as listed in Table 3.

**Table 3:** Mean of maximum concentration (ng/L) (± S.E.), detection frequency (DF) percentage, and relative abundance (R.A.) percentage of pharmaceuticals detected in the influent and effluent wastewater samples

Pharmaceutical class	Pharmaceutical compound	Compounds code	Influent (ng/L)	Effluent (ng/L)	D.F. (%)	R.A. (%) influent urban	R.A. (%) effluent urban	R.A. (%) influent rural	R.A. (%) effluent rural
Antimicrobial (aminoglycoside)	Erythromycin	ERY	ND	BLQ (< 46)	25	BLQ (< 46)	BLQ (< 46)	ND	BLQ (< 46)
Antimicrobial (nitroimidazole)	Metronidazole	MNZ	BLQ (< 20)	BLQ (< 20)	25	BLQ (< 20)	BLQ (< 20)	ND	ND
Antimicrobial (aminopyrimidine)	Trimethoprim	TMP	ND	ND	ND	ND	ND	ND	ND
Antihyperglycemic	Gliclazide	GLZ	BLQ (< 91)	BLQ (< 91)	38	BLQ (< 91)	BLQ (< 91)	BLQ (< 91)	BLQ (< 91)
Antihyperglycemic	Metformin	MTF	660 ± 1	1100 ± 70	88	26	40	25	1
Antilipemic (statin)	Atorvastatin	ATS	BLQ (< 52)	BLQ (< 52)	38	BLQ (< 52)	BLQ (< 52)	ND	ND
Antilipemic (HMG-CoA reductase inhibitors (statins))	Simvastatin	SVS							ND
Antihypertensive, antiarrhythmic	Atenolol	ATN	110 ± 3	130 ± 2	100	2	5	11	3
Antihypertensive, antineoplastic	Captopril	CTP	560 ± 11	ND	13	11	ND	ND	ND
Antihypertensive	Lisinopril	LSP	115 ± 1	33 ± 3	100	4	2	2	14
Antihypertensive, antiarrhythmic, antianginal	Propranolol	PNL	ND	ND	ND	ND	ND	ND	ND
Urological agent, antihypertensive, antianginal	Sildenafil	SDF	BLQ (< 53)	ND	13	BLQ (< 53)	BLQ (< 53)	BLQ (< 53)	BLQ (< 53)
Antidepressants	Amitriptyline	AMT	510 ± 1	BLQ (< 128)	13	12	0.68	ND	BLQ (< 128)
Antihistamine	Chlorpheniramine maleate	CPM	ND	ND	ND	ND	ND	ND	ND
Antihistamine	Diphenhydramine	DPH	BLQ (< 81)	ND	13	BLQ (< 81)	BLQ (< 81)	BLQ (< 81)	BLQ (< 81)
Antispasmodic	Hyoscine butyl bromide	HBB	ND	ND	ND	ND	ND	ND	ND
Non-steroidal anti-inflammatory drug (NSAID)	Mefenamic Acid	MFA	630 ± 3	275 ± 8	100	8	24	53	9
Analgesics, antipyretic	Paracetamol (acetaminophen)	PCM	1200 ± 8	1300 ± 8	88	31	67	6	ND
Antimicrobial (fluoroquinolone)	Ofloxacin	OFX	ND	ND	ND	ND	ND	ND	ND
		**Highest conc. (ng/L)**			**Highest R.A. (%)**				
		1st highest	1200 ± 8 (PCM)	1300 ± 8 (PCM)	1st highest	31 (PCM)	67 (PCM)	53 (MFA)	14 (LSP)
		2nd highest	660 ± 1 (MTF)	1100 ± 70 (MTF)	2nd highest	26 (MTF)	40 (MTF)	25 (MTF)	9 (MFA)
		3rd highest	630 ± 3 (MFA)	275 ± 8 (MFA)	3rd highest	12 (AMT)	23.7 (MFA)	11 (ATN)	3 (ATN)
		4th highest	560 ± 11 (CTP)	130 ± 2 (ATN)	4th highest	11 (CTP)	5 (ATN)	6 (PCM)	1.0 (MTF)
		5th highest	510 ± 1 (AMT)	< LOQ	5th highest	8 (MFA)	2 (LSP)	2 (LSP)	

*S.E.*, standard errors.

*ND*, not detected (< LOD).

BLQ: < LOQ.

DF (%) = (number of WWTPs where the compound was detected/total number of WWTPs) × 100.

R.A. (%) = (total concentration of pharmaceutical “*x*”/total concentrations of all pharmaceuticals) × 100.

The analysis of pharmaceutical concentrations and their relative abundance percentages (R.A.) revealed distinct differences between urban and rural WWTP influent and effluent samples (Table 3). The R.A. of pharmaceutical *x* was calculated by dividing the total concentration of pharmaceutical *x* by the total concentrations of all pharmaceuticals and then multiplying by 100, as expressed in the equation: R.A. (%) = (total concentration of pharmaceutical *x* / total concentrations of all pharmaceuticals) × 100. In urban influents, the highest relative abundances were observed for PCM at 31% and MTF at 26%. In comparison, rural influents exhibited a similar concentration of MTF at 25% and higher concentrations of MFA at 53%. Urban effluents, similar to urban influents, showed elevated abundances of PCM (67%) and MTF (40%), whereas rural effluents had significant concentrations of LSP at 14% and MFA at 9%. These results highlight the variability in pharmaceutical contamination between urban and rural environments, emphasising the need for targeted monitoring and management strategies in various environments.

**Fig. 3:**
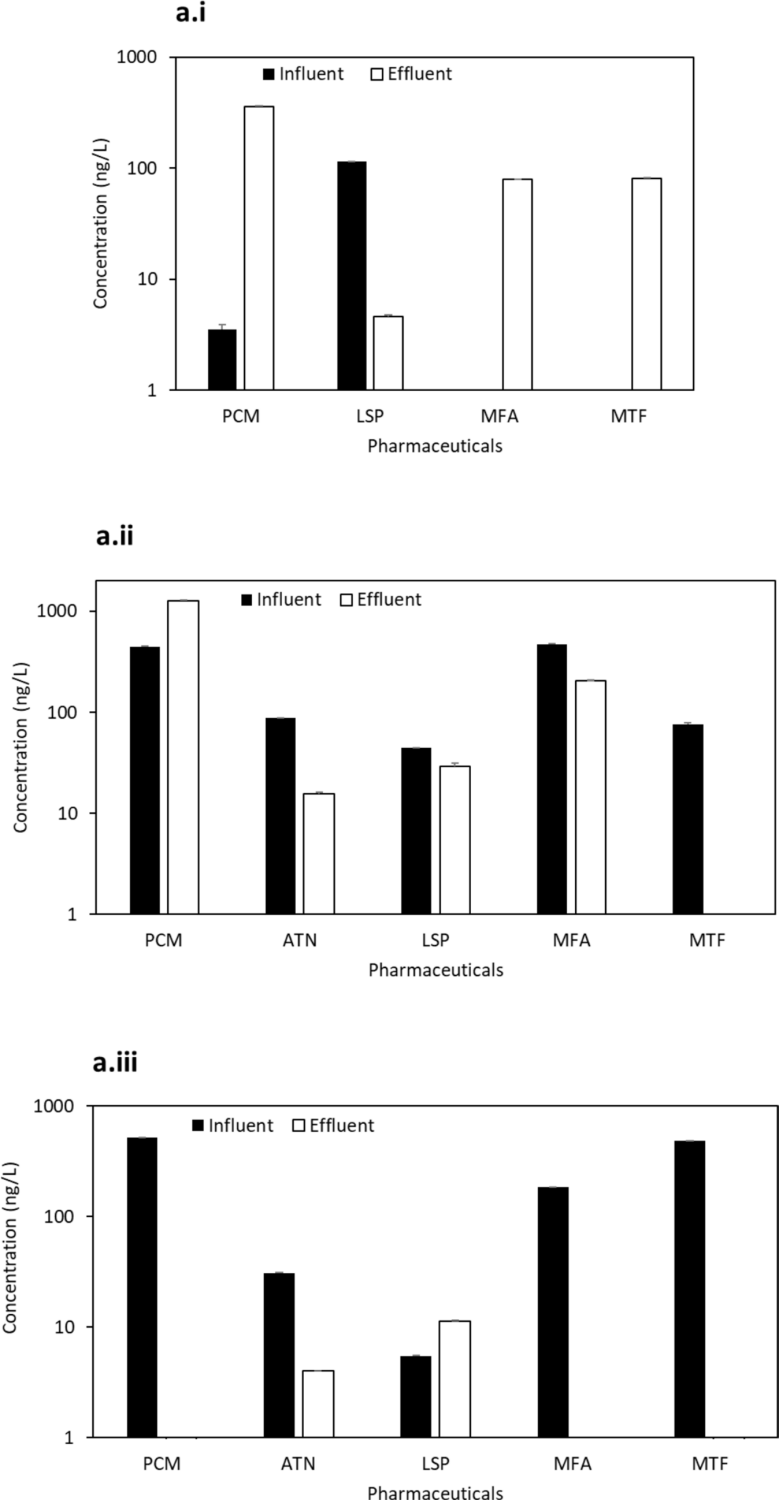
Concentration (ng/L) (mean ± S.E.) of pharmaceuticals at influent and effluent samples from the urban WWTP “**a**” (coupled with MBR treatment system) across 3 days: day 1 (**a.i**), day 2 (**a.ii**), and day 3 (**a.iii**)

Site “a” WWTP is situated in an urban area, coupled with an MBR treatment system, similar to the reported WWTP2-1in Saudi Arabia (Alidina et al. 2014), with a capacity of 125,000 m^3^/day. Our sampling occurred over 3 days during the same season, initiated on a day following heavy rainfall accompanied by tropical cyclone Shaheen. Across the 3 days, a total of seven pharmaceuticals were detected (Table S9), with four compounds (PCM, LSP, MFA, and MTF) present in all three samples (refer to Fig. 3). ATN, which was previously reported in the WWTP2-1 in Saudi Arabia, was also identified in our samples. PCM, MFA, and MTF exhibited varying trends throughout the 3-day study. Specifically, these compounds were found to accumulate in the effluent on day one (Fig. 3 a.i), whereas, by day three, the concentrations detected in the influent were effectively removed following treatment (Fig. 3 a.iii).

**Fig. 4:**
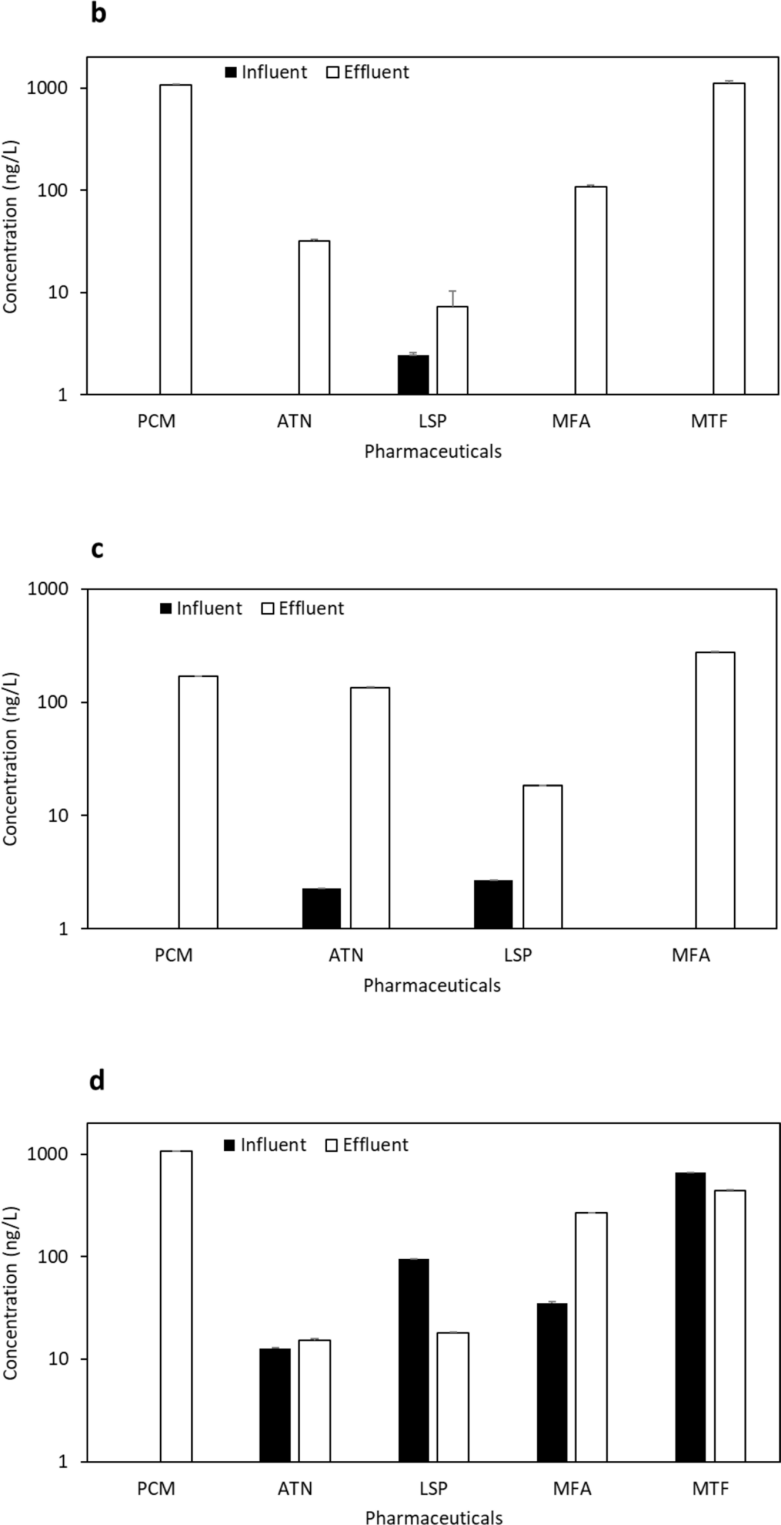

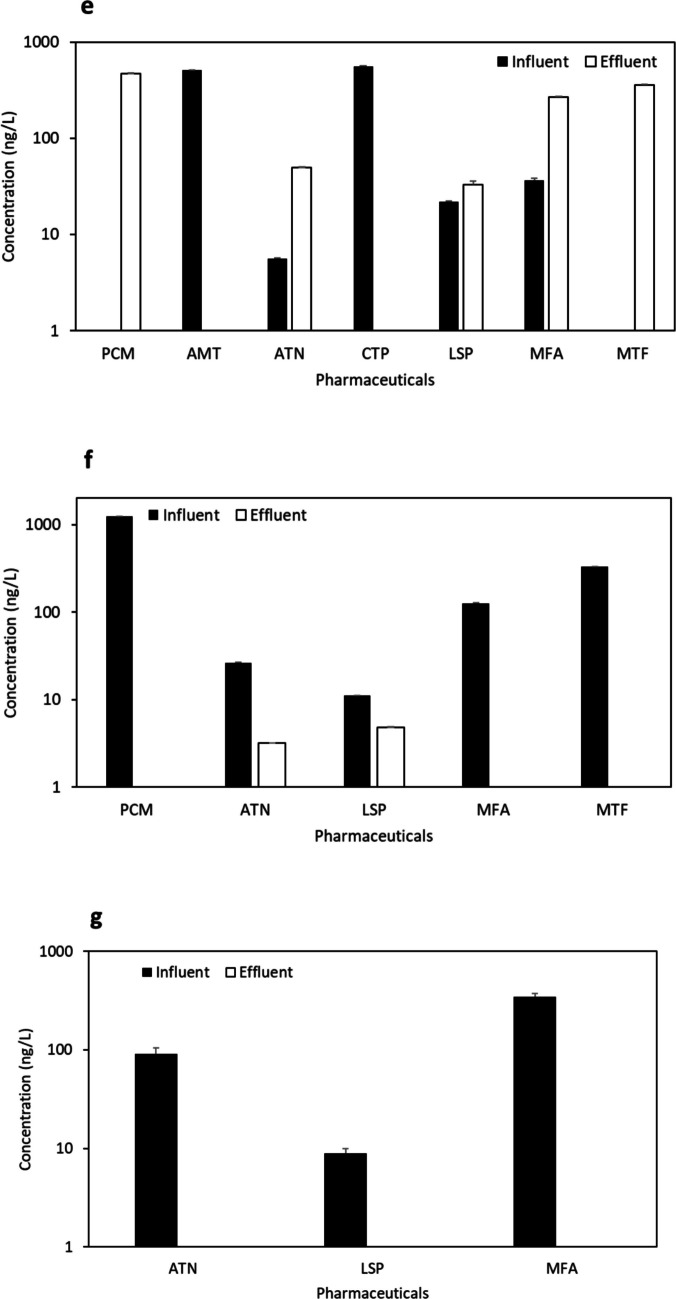

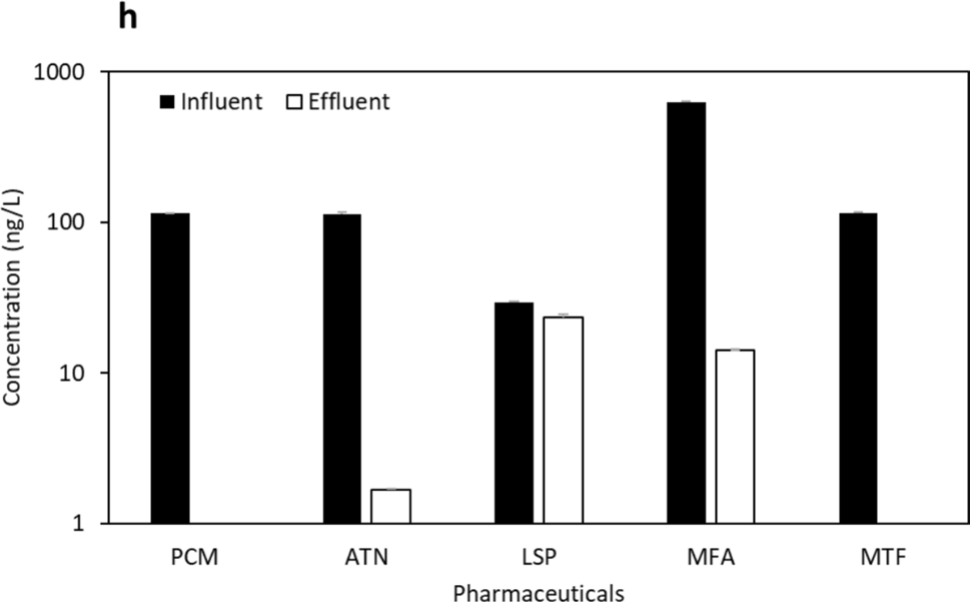
Concentration (ng/L) (mean ± S.E.) of pharmaceuticals at influent and effluent samples from urban and rural WWTPs “**b**, **c**, **d**, **e**, **f**, and **g**” with various treatment processes (see Table S1)

Urban WWTPs at sites “b” and “c”, located in the same city but not in proximity, exhibited four common pharmaceuticals (PCM, ATN, LSP, and MFA) (Fig. 4 b and 4 c) out of the total nine compounds detected (Table S9). The results obtained in site “b”, an aging facility with an MBR treatment system (37,500 m^3^/day capacity), shared similarities with site “c”, which employed a conventional treatment system (9300 m^3^/day capacity). Notably, except for LSP and ATN, the most abundant pharmaceuticals in both sites originated from effluent samples, indicating the release of trace pharmaceuticals below the LOD and accumulation over time into the sites.

Two other urban WWTPs (“d” and “e”) in the same city, possessing similar demographics and capacities (3500 m^3^/day and 2600 m^3^/day, respectively), implemented conventional treatment processes. These sites exhibited five common compounds (PCM, ATN, LSP, MFA, and MTF) (Fig. 4 d and Fig. 4 e). The conventional treatment process has been studied in the MENA region in Saudi Arabia (KSA), Iran, and Tunisia. In two hospital WWTPs in Saudi Arabia, Al Qarni et al. (2016) reported the presence of PCM and ATN. Similarly, Moslah et al. (2018) reported the presence of PCM and ATN in seven WWTPs in Tunisia. Furthermore, site “f”, located in a distinct urban city and utilising an SBR System plus U.F. treatment process with a capacity of 62,500 m^3^/day, shared all the same compounds as sites “d” and “e” (Fig. 4 f).

The final two sites, rural WWTPs with the lowest capacities in the study, included site “g” from an interior region (conventional treatment, 2200 m^3^/day capacity) and site “h” in a coastal region (MBR treatment system, 1000 m^3^/day capacity). Both sites exhibited three common pharmaceuticals (ATN, LSP, and MFA) (Fig. 4 g and Fig. 4 h). Regarding the detected antimicrobials, ERY (aminoglycoside, DF = 25%) and MNZ (nitroimidazole, DF = 25%) were found in various locations but were generally below LOQ. TMP (aminopyrimidine) and OFX (fluoroquinolone) were undetected. In previous research conducted in the MENA region, the concentration of ERY reached 5200 ng/L in HWW influents and 2000 ng/L and 170 ng/L in urban domestic WWTP influents and effluents, respectively (Al-Maadheed et al. 2019). Additionally, Mydlarczyk et al. (2022) reported the maximum MNZ concentration in Kuwait at 45,800 ng/L in HWW influent, while Al-Maadheed et al. (2019) found it in Qatar to be 5500 ng/L in HWW influent and 3100 ng/L and 330 ng/L in urban domestic WWTP influents and effluents, correspondingly. For TMP, the maximum reported concentration was 110 ng/L in the HWW influent in Kuwait (Mydlarczyk et al. 2022), 130 ng/L in the WWTP influent in Jordan (Tahrani et al. 2016), and 785 ng/L in WWTP effluent in Saudi Arabia (Alidina et al. 2014). OFX was detected with a trace amount in Tunisian WWTPs with a maximum detected concentration of 870 ng/L in influent and 650 ng/L in effluent (Moslah et al. 2018). Other antimicrobials were reported in several previous studies conducted in the MENA region (not in ours), including sulfapyridine, sulfamethazine, sulfadiazine, sulfamethoxazole, and ciprofloxacin, as indicated in Table 1.

Antihyperglycemic compounds, including GLZ and MTF, were detected in various WWTPs in this study (DF = 38% and 88%, respectively). While GLZ was detected below the LOQ, MTF emerged as the second highest detected pharmaceutical in influent and effluent, with concentrations recorded at 660 ± 1 ng/L in influent and accumulating to 1100 ± 70 ng/L in effluent. In the MENA region, and among several prior research, hypoglycemics were rarely studied, and MTF has been reported with a maximum of 31 ng/L and 5 ng/L in Saudi Arabian influent and effluent WWTPs, respectively (Shraim et al. 2017), which are much lower than the maximum concentrations detected in this study. Regarding antihypertensive compounds, ATN and LSP were identified in the current work in every WWTP (DF = 100%). ATN exhibited peak concentrations of 110 ± 3 ng/L in influent and 130 ± 2 ng/L in effluent, while LSP showed peak concentrations of 115 ± 1 ng/L in influent and 33 ± 3 ng/L in effluent. CTP (DF = 13%) was undetected in effluent, yet it ranked as the 4th highest detected compound in influent at 560 ± 11 ng/L. PNL was undetected in this study. ATN has been extensively studied in previous research and was detected in Tunisia at 2200 ng/L and 1200 ng/L in WWTP influent and effluent, respectively (Moslah et al. 2018). In Saudi Arabia, it reached concentrations of 730 ng/L in HWW influent (Al Qarni et al. 2016), 4 ng/L in domestic WWTP influent, and 2 ng/L (Shraim et al. 2017) to 2,600 ng/L (Alidina et al. 2014) in WWTP effluent. Thus, certain concentrations of ATN detected in prior studies were lower than those observed in the current work.

In addition, the antidepressant AMT was identified only in the influent at site “e” (DF = 13%) in this study, with the fifth highest concentration recorded in the influent at 510 ± 1 ng/L. In the MENA region, AMT was infrequently studied, and it was found in Saudi Arabia with a maximum concentration of 365 ng/L in WWTP effluent (Alidina et al. 2014). Similarly, the antihistamine DPH was exclusively detected in site “e” (DF = 13%) in the current work, with concentrations below the LOQ. Previous studies in Saudi Arabia reported DPH concentrations of up to 6700 ng/L in effluents (Alidina et al. 2014), whereas in Jordan, DPH was below the LOD (Tahrani et al. 2016). The other antihistamine, CPM, was undetected.

PCM (DF = 88%) and MFA (DF = 100%) were found in all sites, except site “g” for PCM, with the first and third highest concentrations observed in this study, respectively. PCM showed maximum concentrations of 1200 ± 8 ng/L in influent and 1300 ± 8 ng/L in effluent, while MFA exhibited maximum concentrations of 630 ± 3 ng/L in influent and 275 ± 8 ng/L in effluent. In the MENA region, PCM was extensively studied and found in Kuwait, UAE, Tunisia, and Saudi Arabia with the highest detected concentrations of 179,000 ng/L in Kuwaiti HWW influent (Mydlarczyk et al. 2022), 147,000 ng/L in UAE WWTP influent, and 6600 ng/L in UAE WWTP effluent (Semerjian et al. 2018). Antilipemic (statins) SVS was undetected, while ATS was detected at sites “a”, “c”, and “d” (DF = 38), although the concentrations were below the LOQ. Similarly, the urological agent SDF was detected only at site “h” (DF = 13%) but with a concentration below the LOQ.

**Fig. 5:**
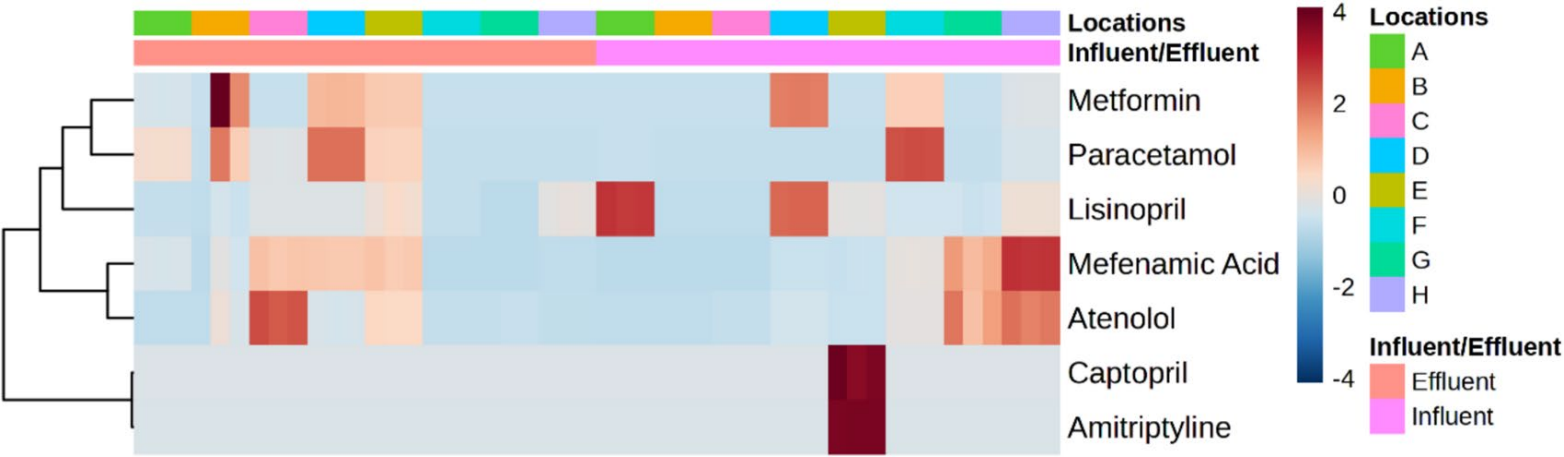
Relative abundance heatmap of 48 samples significantly different (identified by one-way ANOVA, *α* < 0.05) across studied WWTP locations, sample points (influent/effluent), and detected pharmaceuticals in wastewater and treated water. Each row represents a pharmaceutical compound, each column represents a location and sample point (influent/effluent), and the colour of the cells represents the average relative abundance of each pharmaceutical, where red indicates high abundance and blue indicates low abundance

One-way ANOVA (*α* < 0.05) was used for the detected pharmaceuticals in wastewater and treated water across studied WWTP locations and sample points (influent/effluent). A heat map was generated to depict potential variations among multiclass pharmaceuticals (Fig. 5). This data’s hierarchical cluster analysis (HCA) indicates the differentiation into two main groups, with one further subdivided. Each group exhibits a distinct abundance of pharmaceuticals from different classes. The pharmaceutical abundance was generally higher in influent samples compared to effluent samples, with significant concentrations of PCM in site “f”, LSP in site “a”, MFA in site “H” and AMT, and CTP in site “e”. In the effluent samples, high abundances were observed for ATN in site “c”.

Furthermore, this study investigated pharmaceuticals’ removal efficiency (RE) in the eight WWTPs, and information is presented in Table 4. Different treatment methods made distinct contributions to the removal of pharmaceuticals. Most detected pharmaceuticals (69%) exhibited varying removal rates, ranging from partial (9–56%) to significant (78–100%) and complete (100%) removal. Conversely, other pharmaceuticals (31%) displayed negative removal, with higher effluent concentrations than the influent, suggesting their release and accumulation post-treatment in different WWTPs. For instance, GLZ, MTF, ATN, LSP, MFA, and PCM exhibited negative removal in some WWTPs. Nieto-Juarez et al. (2021) have previously reported this phenomenon. In addition, this study had a significant variation in the RE for individual compounds, ranging from − 7000 to 100%. On twenty-one occasions, pharmaceuticals showcased removal rates surpassing 75% across various WWTPs that employed diverse treatment processes.

**Table 4:** Removal efficiency percentage of pharmaceuticals in the eight studied urban and rural WWTPs (sites a, b, c, d, e, f, g, and h)

No	Pharmaceutical compound	Compound code	Site a	Site b	Site c	Site d	Site e	Site f	Site g	Site h
1	Erythromycin	ERY	BLQ	BLQ	BLQ	BLQ	BLQ	ND	ND	BLQ
2	Metronidazole	MNZ	BLQ	BLQ	ND	BLQ	ND	BLQ	ND	ND
3	Trimethoprim	TMP	ND	ND	ND	ND	ND	ND	ND	ND
4	Gliclazide	GLZ	87	ND	− 3000	BLQ	BLQ	BLQ	BLQ	90
5	Metformin	MTF	53	BLQ	BLQ	33	− 7000	98	ND	100
6	Atorvastatin	ATS	100	ND	BLQ	BLQ	ND	ND	ND	ND
7	Simvastatin	SVS	ND	ND	ND	ND	ND	ND	ND	ND
8	Atenolol	ATN	85	BLQ	− 5800	− 21	− 790	88	96	99
9	Captopril	CTP	ND	ND	ND	ND	100	ND	ND	ND
10	Lisinopril	LSP	9	− 200	− 580	81	− 51	56	100	20
11	Propranolol	PNL	ND	ND	ND	ND	ND	ND	ND	ND
12	Sildenafil	SDF	ND	ND	BLQ	BLQ	BLQ	ND	ND	ND
13	Amitriptyline	AMT	ND	ND	BLQ	BLQ	100	ND	ND	ND
14	Chlorpheniramine maleate	CPM	ND	ND	ND	ND	ND	ND	ND	ND
15	Diphenhydramine	DPH	BLQ	ND	BLQ	BLQ	BLQ	ND	ND	BLQ
16	Hyoscine butyl bromide	HBB	ND	ND	ND	ND	BLQ	ND	ND	ND
17	Mefenamic acid	MFA	78	BLQ	BLQ	− 650	− 63	100	100	98
18	Paracetamol (acetaminophen)	PCM	− 3400	BLQ	BLQ	BLQ	BLQ	100	ND	100
19	Ofloxacin	OFX	ND	ND	ND	ND	ND	ND	ND	ND

Removal (%) = ((influent—effluent)/influent) × 100 OR Removal (%) = (1—effluent/influent) × 100.

Note: detected compounds = 65 (48 + ve removal + 17 -ve removal).

*ND*, not detected.

Treatment process-wise, the sites with the MBR process in this study (locations “a, b, and h”) showed significant removal of GLZ, MTF, ATS, ATN, and MFA. In contrast, those utilising the CAS treatment process (sites “c, d, e, and g”) demonstrated significant removal of AMT. These results align with several previous studies that have demonstrated the effectiveness of different treatment processes in removing specific pharmaceuticals. For instance, various studies have reported 100% removal efficiency of PCM in CAS systems (Behera et al. 2011; Gracia-Lor et al. 2012; Nguyen et al. 2018). Similarly, Kasprzyk-Hordern et al. (2008) reported a significant removal efficiency of 100% for PCM and 76% for AMT in a CAS treatment setting. Furthermore, Moslah et al. (2018) highlighted variable removal efficiency for ATN (42 to 59% and − 13 to − 580%), suggesting that the effectiveness of CAS systems can vary significantly.

In addition, extremely negative removal efficiencies were reported in this research for PCM (− 3400% in site “a” but not detected or 100% removed in sites “b and h”) at WWTPs with MBR processes, as well as GLZ (− 3000%), MTF (− 7000%), ATN (− 21 to − 5800%), LSP (− 200%), and MFA (− 63 to − 650%) at WWTPs with CAS processes. In four municipal WWTPs utilising MBR processes in Hawai’i, D’Alessio et al. (2018) reported effective removal rates of ≥ 99% for PCM (consistent with site “h”). Additionally, Kasprzyk-Hordern et al. (2008) found a 74% removal efficiency for MFA, highlighting a discrepancy with the negative removal rates observed at sites “d” and “e” in this study. Behera et al. (2011) identified variable removal efficiencies for ATN (approximately 40%) in five WWTPs coupled with CAS in Ulsan, Korea, while observing poor or no removal of MFA, similar to the current findings.

Interestingly, the SBR plus U.F. treatment process at the only site “f” showed no negative removal efficiency, with most removal efficiencies for various pharmaceuticals above 88%, indicating its effectiveness. Mat Zaini et al. (2022) found that the SBR system is effective in removing ATN (83%) but exhibits partial removal of GLZ (41%). Kolecka et al. (2020) found variable removal of pharmaceuticals, including PCM (52–99%), ibuprofen (− 130–99%), flurbiprofen (ND–100%), naproxen (4–100%), and diclofenac with its metabolites (− 740–100%). These variabilities in the treatment efficiency of various WWTP systems underscore the need for further optimisation of wastewater treatment processes to enhance the removal of pharmaceutical mixtures.

Burcea et al. (2020) pointed out that pharmaceuticals adopt characteristics of persistent organic contaminants. These pharmaceutical compounds are less likely to adsorb to organic material, as indicated by their respective (low) log *K*_ow_ values (0.46, − 1.2, 3.3, 0.16, 3.3, and − 2.6 for PCM, LSP, MFA, ATN, DPH, and MTF, respectively). For instance, after being expelled from the human body, MTF tends to remain in aqueous phases rather than being retained by soil or undergoing sedimentation, which is attributed to its high-water solubility (1000 g/L) and a low log *K*_ow_ of − 2.6 (He et al. 2022).

### Radiopharmaceuticals levels in an urban WWTP

**Fig. 6:**
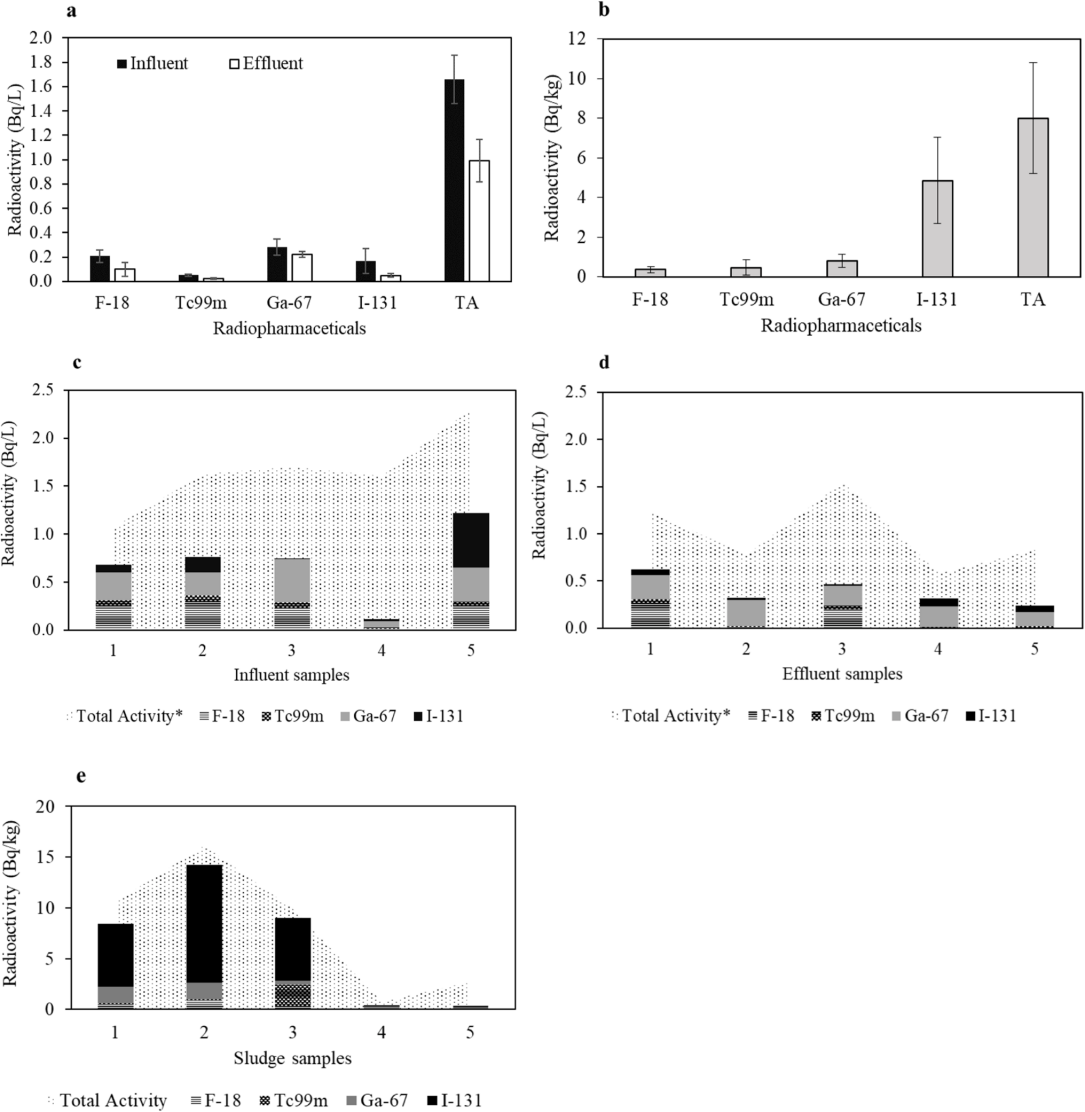
Radioactivity (Bq/L or Bq/kg) (mean ± S.E.) of four radiopharmaceuticals and total activity (T.A.) at **a** an influent and effluent sampling point and **b** sludge samples from a WWTP across a 5-day sampling period. Total activity in comparison with the five samples from **c** influent, **d** effluent wastewater, and **e** sludge. Note: x-axis labels for samples 1–5 correspond to the 5-day sampling period

In this study, the radioactivity of four radiopharmaceuticals and total activity in influent, effluent, and sludge samples from site “a” WWTP across a 5-day sampling period are presented in Table 5 and Fig. 6. The selected radiopharmaceuticals were detected in all samples (DF = 100%). Their activities in influent samples were higher than those in effluent samples (Fig. 6a). Additionally, the total activity in effluents (mean < 1.5 Bq/L; Fig. 6c) observed during our 5-day sampling campaign showed lower radioactivity than influent samples (mean > 1.5 Bq/L; Fig. 6d). Specifically, 18F-FDG, Tc99m, Ga-67, and I-131 exhibited activities of 0.21 ± 0.050 Bq/L, 0.05 ± 0.011 Bq/L, 0.28 ± 0.07 Bq/L, and 0.17 ± 0.10 Bq/L, respectively, in influents, compared to 0.098 ± 0.056 Bq/L, 0.024 ± 0.007 Bq/L, 0.22 ± 0.02 Bq/L, and 0.049 ± 0.015 Bq/L, in effluents. Concerning the removal efficiencies (Table 5), the average removal rates for 18F-FDG and Tc-99 m from wastewater were 51% and 52%, respectively. Conversely, negative average removal efficiencies were observed for I-131 and 67-Ga, reaching − 22% and − 25%, respectively. Moreover, the activity observed in sludge samples (Fig. 6b and e) surpassed that found in wastewater, with 18F-FDG, Tc99m, Ga-67, and I-131 exhibiting activities of 0.37 ± 0.16 Bq/kg, 0.47 ± 0.40 Bq/kg, 0.80 ± 0.33 Bq/kg, and 5 ± 2 Bq/kg. Generally, the total activities in sludge samples were 8 ± 3 Bq/kg compared to 2 ± 0.20 Bq/L and 0.99 ± 0.18 Bq/L in influents and effluents, respectively. I-131 was the highest detected radiopharmaceutical in sludge samples at 5 ± 2 Bq/kg.

**Table 5:** Radioactivity (Bq/L or Bq/kg) (including total activity), detection frequency (D.F.) percentage, and removal efficiency (RE) percentage of radiopharmaceuticals in influent, effluent, and sludge samples from a WWTP across a 5-day sampling period

Radiopharmaceuticals	Compounds code	Influent (Bq/L)			Effluent (Bq/L)			Sludge (Bq/kg)			D.F. (%)	RE (%)
		Mean	Range	SE	Mean	Range	SE	Mean	Range	SE		
18fluorine-fluorodeoxyglucose	F-18	0.21	0.013–0.29	0.050	0.10	0.0056–0.26	0.056	0.37	0.021–0.88	0.16	100	51
Technetium-99 m	Tc99m	0.051	0.013–0.072	0.011	0.024	0.0059–0.46	0.0074	0.47	0.017–2	0.40	100	52
Gallium-67	Ga-67	0.28	0.067–0.45	0.070	0.22	0.14–0.28	0.020	0.80	0.14–2	0.33	100	−25
Iodine-131	I-131	0.17	0.012–0.56	0.10	0.049	0.011–0.085	0.015	5	0.12–12	2	100	−22
Total activity*	TA	2	1–2	0.20	0.99	0.58–2	0.18	8	0.76–16	3	-	-

*Including other isotopes

D.F. (%) = (Number of WWTPs where the compound was detected / Total number of WWTPs) × 100.

Removal (%) = ((influent—effluent) / influent) × 100 OR Removal (%) = (1—effluent/influent) × 100.

Bererhi and Constable (1999) have proposed the utilisation of 100 kBq/kg as the accepted limit whenever the allowance for radioactivity is not set. UK regulations consider this limit the minimum activity concentration for classifying any material as radioactive. Therefore, the overall detected specific activities for Ga-67, F-18, I-131, and Tc-99 m in our study are deemed acceptable, given that they are lower than 100 kBq/kg. Another assumption by Bererhi and Constable (1999) suggested that the specific activity for I-131 in irrigation water should be 5.5 Bq/L, while in sludge, it should be 4 kBq/kg. Consequently, the maximum detected specific activities for I-131 in effluent (0.085 Bq/L) and sludge samples (0.012 kBq/kg) in our study are considered acceptable.

In France, the Decision No. 2008-DC-0095 of the Autorité de Sûreté Nucléaire (French Nuclear Safety Authority) dated 29 January 2008, among other provisions, outlines technical regulations for the discharge of radioactive effluents from hospitals and clinics (IAEA 2010). According to this Decision, the concentration of discharged nuclides to sewers should be less than 10 Bq/kg, or 100 Bq/kg for effluents from rooms of patients treated with I-131. Once more, the maximum specific activities of I-131 (0.085 Bq/L) found in this work align with the regulations set by France. Moreover, Mulas et al. (2019) studied various WWTPs in the Barcelona Metropolitan Area (Spain), including Treatment Plant 5 (TP-5). TP-5 shared similarities with the treatment plant examined in our work, coupled with a comparable treatment process (i.e. MBR) and a similar medium plant size ranging from 40,000 to 80,000 m^3^/dL (site “a” plant size: 125,000 m^3^/day). The specific activities of I-131, Tc-99 m, and 67-Ga in the wastewater were found to be ≤ 0.2, ≤ 5, and ≤ 1 Bq/L, respectively, while in the sludge, these activities were ≤ 5, ≤ 98, and ≤ 19 Bq/L, respectively. Interestingly, these findings parallel the results of our study, with almost all detected activities in our research being lower.

Furthermore, Mulas et al. (2019) suggested that understanding radioactivity levels in waters and sewage sludges could be improved by considering WWTP operational conditions. Notably, there is limited published information on the impact of sewage sludge age, a crucial parameter that varies based on plant conditions and is affected by the short half-lives and decay processes of radiopharmaceuticals used in nuclear medicine procedures. This variability could potentially lead to higher-than-anticipated activities in the eventual solid by-product. Our study encompassed samples obtained from one WWTP over 5 days, aimed at discerning the temporal fluctuations in sewage sludge age. Nonetheless, a longitudinal investigation across multiple WWTPs is advisable. We focused on WWTPs treating municipal wastewater rather than hospital wastewater. Although hospitals typically manage radiopharmaceutical waste separately, residues from patients treated with radiopharmaceuticals can enter municipal sewers through domestic excretion. These contributions are reflected in the signatures detected at WWTPs, highlighting the importance of considering both hospital and community sources in future investigations.

Moreover, radioactive substances, such as iodine-131, are primarily excreted through urine (Barbosa et al. 2022). In the same city as site “a” WWTP in this research, a primary hospital employs two isolation rooms and two subterranean concrete tanks for managing liquid waste generated by patients undergoing nuclear medicine procedures (Ravichandran et al. 2011). This waste is treated using submersible pumps and an ultrasonic system. The system can handle a maximum flow rate of 3.7 MBq/s, maintaining an average monthly concentration of 22 MBq/m^3^ (Ravichandran et al. 2011). Considering potential limitations in hospital capacity, discharging patients after I-131 therapy is a strategic measure to free up hospital beds for other patients. However, this practice raises concerns about releasing contaminated waste into inadequately equipped sewage systems, which are not designed to manage such hazardous waste efficiently. A decade ago, the IAEA indicated that storing urine in delay tanks is unnecessary and that continuous sewage dilutions are satisfactory (Mattsson & Bernhardsson 2012). Considering other factors that play a crucial role in prolonging the specific activity of radiopharmaceuticals, it is pertinent to reevaluate and reconsider this long-standing statement.

## Conclusion

The inevitable presence of pharmaceuticals in the environment via sewage necessitates ongoing monitoring to comprehensively understand their fate within WWTPs and gauge their potential release into water ecosystems. This study addresses and evaluates the challenge of pharmaceutical and radiopharmaceutical contamination in wastewater across the MENA region. The investigation was conducted by analysing influent, effluent, and sludge samples from eight urban and rural WWTPs with diverse treatment processes. The study effectively confirms the presence of thirteen pharmaceuticals and four radiopharmaceuticals in WWTPs in the investigated Arabian Peninsula Nation. Site “a”, an urban WWTP, revealed the presence of seven pharmaceuticals during three days, with four compounds consistently detected. In parallel, two other urban WWTPs (sites “b” and “c”) identified four common pharmaceuticals out of nine, primarily in effluents, indicating gradual accumulation. Furthermore, two additional urban WWTPs (sites “d” and “e”) and a remote urban location (site “f”) with conventional treatment methods shared five compounds. Rural WWTPs (sites “g” and “h”) similarly had three common pharmaceuticals. The total concentrations of the top five pharmaceuticals revealed significant disparities, with higher levels of PCM, MFA, CTP, and AMT in urban wastewater treatment plants, while MTF showed comparable influent levels in both urban and rural settings. The study also investigated radiopharmaceutical presence, revealing Ga-67 predominance in influent and I-131 prevalence in sludge. Sludge samples displayed higher activity levels than wastewater samples, with I-131 being the most detected radiopharmaceutical.

The research highlights the substantial differences in pharmaceutical loads between urban and rural areas, suggesting a need for improved monitoring and regulation in urban wastewater management. Further research is warranted to understand the intricate toxicity of pharmaceutical interactions within wastewater systems prior to recycling and reuse. The study found variabilities in treatment efficiency with negative removal trends for certain emerging pollutants, including PCM, LSP, MFA, ATN, and MTF, underscoring the urgency of their control and elimination during treatment. In addition, the study prompts consideration of correlated factors, including the non-radioactive aspects of radiopharmaceuticals, such as bio-vectors and bifunctional agents. This underscores the need for robust control measures, treatment protocols, standards, and policies to address these emerging pollutants in the region and beyond. Additionally, the study calls for continued exploration of pharmaceutical and radiopharmaceutical behaviour throughout sewage treatment processes and in the effluent-receiving environment, as well as a critical emphasis on monitoring the environmental impact of medical radiopharmaceutical releases. Establishing a pharmaceutical and radiopharmaceutical ecotoxicity toolbox to characterise water quality for recycling is a recommended step forward.

## Supplementary Information

Below is the link to the electronic supplementary material.

Supplementary file1 (DOCX 339 KB)

## Data Availability

The authors declare that the data supporting the findings of this study are available within the paper and its Supplementary Information files. Should any raw data files be needed in another format they are available from the corresponding author upon reasonable request.

## Abbreviations

WWTP(s): Wastewater treatment plant(s)
MENA: Middle East and North Africa
ERY: Erythromycin stearate
MNZ: Metronidazole
OFX: Ofloxacin
TMP: Trimethoprim
GLZ: Gliclazide
MTF: Metformin
ATS: Atorvastatin
SVS: Simvastatin
ATN: Atenolol
CTP: Captopril
LSP: Lisinopril
PNL: Propranolol
SDL: Sildenafil
AMT: Amitriptyline
CPM: Chlorpheniramine maleate
DPH: Diphenhydramine
HBB: Hyoscine butyl bromide
MFA: Mefenamic acid
PCM: Paracetamol
I-131: Iodine-131
18F-FDG: 18Fluorine-fluorodeoxyglucose
Tc-99m: Technetium-99 m
67-Ga: Gallium-67
