# Supplementary material for: Pharmaceuticals and radiopharmaceuticals in wastewater treatment plants: insights from an Arabian Peninsula nation
Source: Environ Sci Pollut Res Int. 2025 Mar 29;32(15):9844–71. doi: 10.1007/s11356-025-36287-6 (PMC11991994; doi:10.1007/s11356-025-36287-6)
Supplement: Supplementary file 1 — Supplementary file1 (DOCX 339 KB) [file 11356_2025_36287_MOESM1_ESM.docx]

**Pharmaceuticals and radiopharmaceuticals in wastewater treatment plants: insights from an Arabian Peninsula nation**

Ali Alfarsi^1,2*^, Anupama Kumar^2^, Abbasher M. Gismelseed^3^, Ahlam Al Azkawi^4#^, Marwa Al Mahdouri^5^, Fadhila N. Al Mabsali^3^, Sathish Babu^4^, Yaqoob Al Harthy^5^, Muna Al Hosni^4^, Dayanthi Nugegoda^1^

^1^RMIT University, Bundoora West Campus, Bundoora, Victoria 3083, Australia

^2^ CSIRO Environment, Waite Campus, Urrbrae, SA 5064, Australia

^3^Department of Physics, College of Science, Sultan Qaboos University, P.O. Box 36, Code 123, Al Khoud, Muscat,Oman

^4^Central Analytical and Applied Research Unit (CAARU), College of Science, Sultan Qaboos University, Code 123, Al Khoud, Muscat, Oman

^5^Oman Water and Wastewater Services Company (OWWSC), Muscat, Oman

*Corresponding Author Email: ali.alfarsi@student.rmit.edu.au; Ali.Alfarsi@csiro.au

^#^ Author has moved since the work described in the article was done.

*** Corresponding Author**

Ali Alfarsi: [s3568820@student.rmit.edu.au](mailto:s3568820@student.rmit.edu.au)

## Supplementary tables

**Table S1** Characteristics of selected WWTPs

| **WWTPs** | **Capacity (m^3^/d)*** | **Type of Treatment** | **Region Type** |
| --- | --- | --- | --- |
| **a** | 125,000 | Membrane Bioreactor (MBR) System | Urban |
| **b** | 37,500 | MBR System | Urban |
| **c** | 9,300 | Conventional Activated Sludge (CAS) | Urban |
| **d** | 3,500 | CAS | Urban |
| **e** | 2,600 | CAS | Urban |
| **f** | 62,500 | Sequencing Batch Reactor (SBR) System + Ultrafiltration (UF) | Urban |
| **g** | 2,200 | CAS | Rural |
| **h** | 1,000 | MBR System | Rural |

*As in 2022

**Table S2** Water Quality of the Studied WWTPs (A-H)

| **Site** | **Sample** | **Id** | **Analysis** | **Component Name** | **Result** | **Units** |
| --- | --- | --- | --- | --- | --- | --- |
| Site A | influent | RT-influent-G-27DEC21-354123 | BOD5_RESP | Biochemical Oxygen Demand (Reading) | 200 | mg/L |
| Site A | influent | RT-influent-G-27DEC21-354123 | COD | Chemical Oxygen Demand (Reading) | 310 | mg/L |
| Site A | influent | RT-influent-G-27DEC21-354123 | TSS | Total Suspended Solids (Reading) | 160 | mg/L |
| Site A | influent | RT-influent-G-27DEC21-354123 | NH3_N_500C | Ammoniacal Nitrogen (Reading) | 35 | mg/L |
| Site A | influent | RT-influent-G-27DEC21-354123 | TP | Phosphorus Total as P (Reading) | 3 | mg/L |
| Site A | influent | RT-influent-G-27DEC21-354123 | EC | Electrical Conductivity @ 25 °C | 5,500 | uS/cm |
| Site A | influent | RT-influent-G-27DEC21-354123 | TDS_EC | Electrical Conductivity @ 25 °C | 5,500 | uS/cm |
| Site A | influent | RT-influent-G-27DEC21-354123 | TDS_EC | Total Dissolved Solids (TDS by EC Method | 3,300 | mg/L |
| Site A | influent | RT-influent-G-27DEC21-354123 | ALKALINITY | Alkalinity Total as CaCO3 (Reading) | 280 | mg/L |
| Site A | influent | RT-influent-G-27DEC21-354123 | FOG_AQ | Oil and Grease (Reading) | 16 | mg/L |
| Site A | influent | RT-influent-G-27DEC21-354123 | TKN_500C | Total Kjeldahl Nitrogen (Reading) | 26 | mg/L |
| Site A | effluent | RT-effluent-C-27DEC21-354114 | COD | Chemical Oxygen Demand (Reading) | 17 | mg/L |
| Site A | effluent | RT-effluent-C-27DEC21-354114 | TSS | Total Suspended Solids (Reading) | 2 | mg/L |
| Site A | effluent | RT-effluent-C-27DEC21-354114 | TP | Phosphorus Total as P (Reading) | 3 | mg/L |
| Site A | effluent | RT-effluent-C-27DEC21-354114 | NH3_N_500C | Ammoniacal Nitrogen (Reading) | 0.2 | mg/L |
| Site A | effluent | RT-effluent-C-27DEC21-354114 | ANIONS | NO3 (Reading) | 25 | mg/L |
| Site A | effluent | RT-effluent-C-27DEC21-354114 | ANIONS | NO3_N | 6 | mg/L |
| Site A | effluent | RT-effluent-C-27DEC21-354114 | EC | Electrical Conductivity @ 25 °C | 3,960 | uS/cm |
| Site A | effluent | RT-effluent-C-27DEC21-354114 | TDS_EC | Electrical Conductivity @ 25 °C | 3,960 | uS/cm |
| Site A | effluent | RT-effluent-C-27DEC21-354114 | TDS_EC | Total Dissolved Solids (TDS by EC Method | 2,370 | mg/L |
| Site A | effluent | RT-effluent-C-27DEC21-354114 | TKN_500C | Total Kjeldahl Nitrogen (Reading) | 0.6 | mg/L |
| Site A | effluent | RT-effluent-C-27DEC21-354114 | SALINITY | Salinity | 2 | psu |
| Site A | effluent | RT-effluent-C-27DEC21-354114 | BOD5 | Biochemical Oxygen Demand (Avg) | 2 | mg/L |
| Site A | effluent | RT-effluent-G-27DEC21-354116 | VHO | Ova per liter | <1 | Ova/L |
| Site A | effluent | RT-effluent-G-27DEC21-354116 | PH_AQ | pH | 8 | pH_unit |
| Site A | effluent | RT-effluent-G-27DEC21-354116 | PH_AQ | Temperature | 27 | deg_C |
| Site A | effluent | RT-effluent-G-27DEC21-354116 | CL2_RES | Chlorine Residual as Cl2 (Reading) | 2 | mg/L |
| Site A | effluent | RT-effluent-G-27DEC21-354116 | FOG_AQ | Oil and Grease (Reading) | 0.2 | mg/L |
| Site A | effluent | RT-effluent-G-27DEC21-354116 | COLI_FC_MF | Thermotolerant Fecal Coliform | 1 | cfu/100ml |
| Site A | effluent | RT-effluent-G-28DEC21-354204 | COLI_FC_MF | Thermotolerant Fecal Coliform | 1 | cfu/100ml |
| Site A | effluent | RT-effluent-C-29DEC21-354296 | ANION_HACH | NO2 (Reading) | 16 | mg/L |
| Site A | effluent | RT-effluent-C-29DEC21-354296 | ANION_HACH | NO2_N | 4 | mg/L |
| Site A | effluent | RT-effluent-C-29DEC21-354296 | ANION_HACH | NO3 (Reading) | 19 | mg/L |
| Site A | effluent | RT-effluent-C-29DEC21-354296 | ANION_HACH | NO3_N | 4 | mg/L |
|  |  |  |  |  |  |  |
| Site B | influent | RT-influent-C-28DEC21-354205 | COD | Chemical Oxygen Demand (Reading) | 510 | mg/L |
| Site B | influent | RT-influent-C-28DEC21-354205 | NH3_N_500C | Ammoniacal Nitrogen (Reading) | 21 | mg/L |
| Site B | influent | RT-influent-C-28DEC21-354205 | BOD5_RESP | Biochemical Oxygen Demand (Reading) | 210 | mg/L |
| Site B | influent | RT-influent-C-28DEC21-354205 | TKN_500C | Total Kjeldahl Nitrogen (Reading) | 36 | mg/L |
| Site B | influent | RT-influent-C-28DEC21-354205 | TSS | Total Suspended Solids (Reading) | 69 | mg/L |
| Site B | influent | RT-influent-C-28DEC21-354205 | TP | Phosphorus Total as P (Reading) | 8 | mg/L |
| Site B | influent | RT-influent-G-28DEC21-354216 | ALKALINITY | Alkalinity Total as CaCO3 (Reading) | 210 | mg/L |
| Site B | influent | RT-influent-G-28DEC21-354216 | FOG_AQ | Oil and Grease (Reading) | 17 | mg/L |
| Site B | effluent | RT-effluent-C-28DEC21-354197 | COD | Chemical Oxygen Demand (Reading) | 17 | mg/L |
| Site B | effluent | RT-effluent-C-28DEC21-354197 | TSS | Total Suspended Solids (Reading) | 7 | mg/L |
| Site B | effluent | RT-effluent-C-28DEC21-354197 | TP | Phosphorus Total as P (Reading) | 0.12 | mg/L |
| Site B | effluent | RT-effluent-C-28DEC21-354197 | BOD5 | Biochemical Oxygen Demand (Avg) | 6 | mg/L |
| Site B | effluent | RT-effluent-C-28DEC21-354197 | NH3_N_500C | Ammoniacal Nitrogen (Reading) | 0.25 | mg/L |
| Site B | effluent | RT-effluent-C-28DEC21-354197 | TKN_500C | Total Kjeldahl Nitrogen (Reading) | 0.49 | mg/L |
| Site B | effluent | RT-effluent-C-28DEC21-354197 | ANION_HACH | NO2 (Reading) | 0.02 | mg/L |
| Site B | effluent | RT-effluent-C-28DEC21-354197 | ANION_HACH | NO2_N | 0.01 | mg/L |
| Site B | effluent | RT-effluent-C-28DEC21-354197 | ANION_HACH | NO3 (Reading) | 5 | mg/L |
| Site B | effluent | RT-effluent-C-28DEC21-354197 | ANION_HACH | NO3_N | 1 | mg/L |
| Site B | effluent | RT-effluent-G-28DEC21-354201 | PH_AQ | pH | 7 | pH_unit |
| Site B | effluent | RT-effluent-G-28DEC21-354201 | PH_AQ | Temperature | 19 | deg_C |
| Site B | effluent | RT-effluent-G-28DEC21-354201 | CL2_RES | Chlorine Residual as Cl2 (Reading) | 0.85 | mg/L |
| Site B | effluent | RT-effluent-G-28DEC21-354201 | FOG_AQ | Oil and Grease (Reading) | 0.47 | mg/L |
| Site B | effluent | RT-effluent-G-28DEC21-354201 | COLI_FC_MF | Thermotolerant Fecal Coliform | <1 | cfu/L |
| Site B | effluent | RT-effluent-G-28DEC21-354201 | VHO | Ova per liter | <1 | Ova/L |
| Site B | effluent | RT-effluent-G-29DEC21-354304 | T_CL | Total Chlorine (Reading) | 1 | mg/L |
| Site B | effluent | RT-effluent-G-05JAN22-354830 | VNO | Ova per liter | <1.000 | Ova/L |
|  |  |  |  |  |  |  |
| Site C | influent | RT-influent-C-02JAN22-354638 | COD | Chemical Oxygen Demand (Reading) | 1,290 | mg/L |
| Site C | influent | RT-influent-C-02JAN22-354638 | TSS | Total Suspended Solids (Reading) | 740 | mg/L |
| Site C | influent | RT-influent-C-02JAN22-354638 | TP | Phosphorus Total as P (Reading) | 15 | mg/L |
| Site C | influent | RT-influent-C-02JAN22-354638 | NH3_N_500C | Ammoniacal Nitrogen (Reading) | 67 | mg/L |
| Site C | influent | RT-influent-C-02JAN22-354638 | TKN_500C | Total Kjeldahl Nitrogen (Reading) | 100 | mg/L |
| Site C | influent | RT-influent-C-02JAN22-354638 | BOD5_RESP | Biochemical Oxygen Demand (Reading) | 400 | mg/L |
| Site C | influent | RT-influent-G-02JAN22-354642 | ALKALINITY | Alkalinity Total as CaCO3 (Reading) | 470 | mg/L |
| Site C | influent | RT-influent-G-02JAN22-354642 | FOG_AQ | Oil and Grease (Reading) | 23 | mg/L |
| Site C | effluent | RT-effluent-C-02JAN22-354635 | COD | Chemical Oxygen Demand (Reading) | 28 | mg/L |
| Site C | effluent | RT-effluent-C-02JAN22-354635 | TSS | Total Suspended Solids (Reading) | 7 | mg/L |
| Site C | effluent | RT-effluent-C-02JAN22-354635 | TP | Phosphorus Total as P (Reading) | 4 | mg/L |
| Site C | effluent | RT-effluent-C-02JAN22-354635 | NH3_N_500C | Ammoniacal Nitrogen (Reading) | 16 | mg/L |
| Site C | effluent | RT-effluent-C-02JAN22-354635 | TKN_500C | Total Kjeldahl Nitrogen (Reading) | 18 | mg/L |
| Site C | effluent | RT-effluent-C-02JAN22-354635 | BOD5_RESP | Biochemical Oxygen Demand (Reading) | 14 | uS/cm |
| Site C | effluent | RT-effluent-C-02JAN22-354635 | ANION_HACH | NO2 (Reading) | 0.03 | mg/L |
| Site C | effluent | RT-effluent-C-02JAN22-354635 | ANION_HACH | NO2_N | 0.01 | mg/L |
| Site C | effluent | RT-effluent-C-02JAN22-354635 | ANION_HACH | NO3 (Reading) | 18 | mg/L |
| Site C | effluent | RT-effluent-C-02JAN22-354635 | ANION_HACH | NO3_N | 4 | mg/L |
| Site C | effluent | RT-effluent-G-02JAN22-354637 | PH_AQ | pH | 8 | mg/L |
| Site C | effluent | RT-effluent-G-02JAN22-354637 | PH_AQ | Temperature | 26 | mg/L |
| Site C | effluent | RT-effluent-G-02JAN22-354637 | CL2_RES | Chlorine Residual as Cl2 (Reading) | 2 | mg/L |
| Site C | effluent | RT-effluent-G-02JAN22-354637 | FOG_AQ | Oil and Grease (Reading) | 0.29 | mg/L |
| Site C | effluent | RT-effluent-G-02JAN22-354637 | COLI_FC_MF | Thermotolerant Fecal Coliform | 2 | mg/L |
| Site C | effluent | RT-effluent-G-02JAN22-354637 | VHO | Ova per liter | 4 | Ova/L |
|  |  |  |  |  |  |  |
| Site D | influent | RT-influent-C-02JAN22-354643 | BOD5_RESP | Biochemical Oxygen Demand (Reading) | 290 | mg/L |
| Site D | influent | RT-influent-C-02JAN22-354643 | COD | Chemical Oxygen Demand (Reading) | 1,100 | mg/L |
| Site D | influent | RT-influent-C-02JAN22-354643 | TSS | Total Suspended Solids (Reading) | 1,020 | mg/L |
| Site D | influent | RT-influent-C-02JAN22-354643 | NH3_N_500C | Ammoniacal Nitrogen (Reading) | 35 | mg/L |
| Site D | influent | RT-influent-C-02JAN22-354643 | TP | Phosphorus Total as P (Reading) | 15 | mg/L |
| Site D | influent | RT-influent-C-02JAN22-354643 | SALINITY | Salinity | 0.8 | psu |
| Site D | influent | RT-influent-C-02JAN22-354643 | EC | Electrical Conductivity @ 25 °C | 1,980 | uS/cm |
| Site D | influent | RT-influent-C-02JAN22-354643 | TKN_500C | Total Kjeldahl Nitrogen (Reading) | 90 | mg/L |
| Site D | influent | RT-influent-C-02JAN22-354643 | ANION_HACH | NO3 (Reading) | 2 | mg/L |
| Site D | influent | RT-influent-C-02JAN22-354643 | ANION_HACH | NO3_N | 0.39 | mg/L |
| Site D | influent | RT-influent-G-02JAN22-354672 | FOG_AQ | Oil and Grease (Reading) | 22 | mg/L |
| Site D | influent | RT-influent-G-02JAN22-354672 | ALKALINITY | Alkalinity Total as CaCO3 (Reading) | 220 | mg/L |
| Site D | effluent | RT-effluent-C-02JAN22-354644 | BOD5_RESP | Biochemical Oxygen Demand (Reading) | 6 | mg/L |
| Site D | effluent | RT-effluent-C-02JAN22-354644 | COD | Chemical Oxygen Demand (Reading) | 24 | mg/L |
| Site D | effluent | RT-effluent-C-02JAN22-354644 | TSS | Total Suspended Solids (Reading) | 2 | mg/L |
| Site D | effluent | RT-effluent-C-02JAN22-354644 | NH3_N_500C | Ammoniacal Nitrogen (Reading) | 0.54 | mg/L |
| Site D | effluent | RT-effluent-C-02JAN22-354644 | TP | Phosphorus Total as P (Reading) | 3 | mg/L |
| Site D | effluent | RT-effluent-C-02JAN22-354644 | EC | Electrical Conductivity @ 25 °C | 1,890 | uS/cm |
| Site D | effluent | RT-effluent-C-02JAN22-354644 | SALINITY | Salinity | 0.73 | psu |
| Site D | effluent | RT-effluent-C-02JAN22-354644 | TKN_500C | Total Kjeldahl Nitrogen (Reading) | 1 | mg/L |
| Site D | effluent | RT-effluent-C-02JAN22-354644 | ANION_HACH | NO2 (Reading) | 0.083 | mg/L |
| Site D | effluent | RT-effluent-C-02JAN22-354644 | ANION_HACH | NO2_N | 0.027 | mg/L |
| Site D | effluent | RT-effluent-C-02JAN22-354644 | ANION_HACH | NO3 (Reading) | 6 | mg/L |
| Site D | effluent | RT-effluent-C-02JAN22-354644 | ANION_HACH | NO3_N | 1 | mg/L |
| Site D | effluent | RT-effluent-C-02JAN22-354644 | TN_CAL_HAC | Total Nitrogen (Calculated) | 3 | mg/L |
| Site D | effluent | RT-effluent-G-02JAN22-354673 | CL2_RES | Chlorine Residual as Cl2 (Reading) | 0.26 | mg/L |
| Site D | effluent | RT-effluent-G-02JAN22-354673 | FOG_AQ | Oil and Grease (Reading) | <0.15 | mg/L |
| Site D | effluent | RT-effluent-G-02JAN22-354673 | COLI_FC_MF | Thermotolerant Fecal Coliform | <1 | cfu/100ml |
| Site D | effluent | RT-effluent-G-02JAN22-354673 | T_CL | Total Chlorine (Reading) | 0.48 | mg/L |
| Site D | effluent | RT-effluent-G-05JAN22-354858 | VHO | Ova per liter | <1 | Ova/L |
|  |  |  |  |  |  |  |
| Site E | effluent | EXTERNAL-14FEB22-358929 | BOD5 | Biochemical Oxygen Demand (Avg) | <2 | mg/L |
| Site E | effluent | EXTERNAL-14FEB22-358929 | TSS | Total Suspended Solids (Reading) | <0.9 | mg/L |
| Site E | effluent | EXTERNAL-14FEB22-358929 | ANIONS | Chloride (Reading) | 200 | mg/L |
| Site E | effluent | EXTERNAL-14FEB22-358929 | ANIONS | NO3 (Reading) | 0.96 | mg/L |
| Site E | effluent | EXTERNAL-14FEB22-358929 | ANIONS | NO3_N | 0.22 | mg/L |
| Site E | effluent | EXTERNAL-14FEB22-358929 | EC | Electrical Conductivity @ 25 °C | 770 | uS/cm |
| Site E | effluent | EXTERNAL-14FEB22-358929 | PH_AQ | pH | 8 | pH_unit |
| Site E | effluent | EXTERNAL-14FEB22-358929 | PH_AQ | Temperature | 23 | deg_C |
| Site E | effluent | EXTERNAL-14FEB22-358929 | COLI_FC_MF | Thermotolerant Fecal Coliform | <1 | cfu/100ml |
| Site E | effluent | EXTERNAL-14FEB22-358929 | TP | Phosphorus Total as P (Reading) | 0.25 | mg/L |
| Site E | effluent | EXTERNAL-14FEB22-358929 | COD | Chemical Oxygen Demand (Reading) | 57 | mg/L |
| Site E | effluent | EXTERNAL-14FEB22-358929 | TDS_EC | Electrical Conductivity @ 25 °C | 770 | uS/cm |
| Site E | effluent | EXTERNAL-14FEB22-358929 | TDS_EC | Total Dissolved Solids (TDS by EC Method | 460 | mg/L |
| Site E | effluent | EXTERNAL-14FEB22-358929 | FOG_AQ | Oil and Grease (Reading) | <0.15 | mg/L |
| Site E | effluent | EXTERNAL-14FEB22-358929 | NH3_N_500C | Ammoniacal Nitrogen (Reading) | <0.10 | mg/L |
| Site E | effluent | EXTERNAL-14FEB22-358929 | VHO_5L_TP | Total Ova Count / 5L | <1 | No./L |
| Site E | effluent | EXTERNAL-14FEB22-358929 | PHEN_TOTAL | Phenol Total | <0.00080 | mg/L |
|  |  |  |  |  |  |  |
| Site F | effluent | EXTERNAL-15FEB22-359001 | BOD5 | Biochemical Oxygen Demand (Avg) | 3 | mg/L |
| Site F | effluent | EXTERNAL-15FEB22-359001 | TSS | Total Suspended Solids (Reading) | 12 | mg/L |
| Site F | effluent | EXTERNAL-15FEB22-359001 | ANIONS | Chloride (Reading) | 420 | mg/L |
| Site F | effluent | EXTERNAL-15FEB22-359001 | ANIONS | NO3 (Reading) | 0.47 | mg/L |
| Site F | effluent | EXTERNAL-15FEB22-359001 | ANIONS | NO3_N | 0.11 | mg/L |
| Site F | effluent | EXTERNAL-15FEB22-359001 | EC | Electrical Conductivity @ 25 °C | 2,150 | uS/cm |
| Site F | effluent | EXTERNAL-15FEB22-359001 | PH_AQ | pH | 8 | pH_unit |
| Site F | effluent | EXTERNAL-15FEB22-359001 | PH_AQ | Temperature | 24 | deg_C |
| Site F | effluent | EXTERNAL-15FEB22-359001 | COLI_FC_MF | Thermotolerant Fecal Coliform | 270 | cfu/100ml |
| Site F | effluent | EXTERNAL-15FEB22-359001 | TP | Phosphorus Total as P (Reading) | 2 | mg/L |
| Site F | effluent | EXTERNAL-15FEB22-359001 | COD | Chemical Oxygen Demand (Reading) | 75 | mg/L |
| Site F | effluent | EXTERNAL-15FEB22-359001 | TDS_EC | Electrical Conductivity @ 25 °C | 2,150 | uS/cm |
| Site F | effluent | EXTERNAL-15FEB22-359001 | TDS_EC | Total Dissolved Solids (TDS by EC Method | 1,290 | mg/L |
| Site F | effluent | EXTERNAL-15FEB22-359001 | FOG_AQ | Oil and Grease (Reading) | 0.15 | mg/L |
| Site F | effluent | EXTERNAL-15FEB22-359001 | NH3_N_500C | Ammoniacal Nitrogen (Reading) | 42 | mg/L |
| Site F | effluent | EXTERNAL-15FEB22-359001 | VHO_5L_TP | Total Ova Count / 5L | 47 | No./L |
| Site F | effluent | EXTERNAL-15FEB22-359001 | PHEN_TOTAL | Phenol Total | 0.0035 | mg/L |
|  |  |  |  |  |  |  |
| Site G | effluent | EXTERNAL-15FEB22-359006 | BOD5 | Biochemical Oxygen Demand (Avg) | 2 | mg/L |
| Site G | effluent | EXTERNAL-15FEB22-359006 | TSS | Total Suspended Solids (Reading) | <0.9 | mg/L |
| Site G | effluent | EXTERNAL-15FEB22-359006 | ANIONS | Chloride (Reading) | 480 | mg/L |
| Site G | effluent | EXTERNAL-15FEB22-359006 | ANIONS | NO3 (Reading) | 1 | mg/L |
| Site G | effluent | EXTERNAL-15FEB22-359006 | ANIONS | NO3_N | 0.26 | mg/L |
| Site G | effluent | EXTERNAL-15FEB22-359006 | EC | Electrical Conductivity @ 25 °C | 1,450 | uS/cm |
| Site G | effluent | EXTERNAL-15FEB22-359006 | PH_AQ | pH | 8 | pH_unit |
| Site G | effluent | EXTERNAL-15FEB22-359006 | PH_AQ | Temperature | 24 | deg_C |
| Site G | effluent | EXTERNAL-15FEB22-359006 | COLI_FC_MF | Thermotolerant Fecal Coliform | 10 | cfu/100ml |
| Site G | effluent | EXTERNAL-15FEB22-359006 | TP | Phosphorus Total as P (Reading) | 0.62 | mg/L |
| Site G | effluent | EXTERNAL-15FEB22-359006 | COD | Chemical Oxygen Demand (Reading) | 64 | mg/L |
| Site G | effluent | EXTERNAL-15FEB22-359006 | TDS_EC | Electrical Conductivity @ 25 °C | 1,450 | uS/cm |
| Site G | effluent | EXTERNAL-15FEB22-359006 | TDS_EC | Total Dissolved Solids (TDS by EC Method | 870 | mg/L |
| Site G | effluent | EXTERNAL-15FEB22-359006 | FOG_AQ | Oil and Grease (Reading) | <0.15 | mg/L |
| Site G | effluent | EXTERNAL-15FEB22-359006 | NH3_N_500C | Ammoniacal Nitrogen (Reading) | 5 | mg/L |
| Site G | effluent | EXTERNAL-15FEB22-359006 | VHO_5L_TP | Total Ova Count / 5L | <1 | No./L |
| Site G | effluent | EXTERNAL-15FEB22-359006 | PHEN_TOTAL | Phenol Total | 0.00094 | mg/L |
|  |  |  |  |  |  |  |
| Site H | effluent | EXTERNAL-08FEB22-358443 | BOD5 | Biochemical Oxygen Demand (Avg) | <2 | mg/L |
| Site H | effluent | EXTERNAL-08FEB22-358443 | TSS | Total Suspended Solids (Reading) | <0.9 | mg/L |
| Site H | effluent | EXTERNAL-08FEB22-358443 | ANIONS | Chloride (Reading) | 260 | mg/L |
| Site H | effluent | EXTERNAL-08FEB22-358443 | ANIONS | NO3 (Reading) | 0.13 | mg/L |
| Site H | effluent | EXTERNAL-08FEB22-358443 | ANIONS | NO3_N | 0.03 | mg/L |
| Site H | effluent | EXTERNAL-08FEB22-358443 | EC | Electrical Conductivity @ 25 °C | 710 | uS/cm |
| Site H | effluent | EXTERNAL-08FEB22-358443 | PH_AQ | pH | 8 | pH_unit |
| Site H | effluent | EXTERNAL-08FEB22-358443 | PH_AQ | Temperature | 23 | deg_C |
| Site H | effluent | EXTERNAL-08FEB22-358443 | COLI_FC_MF | Thermotolerant Fecal Coliform | <1 | cfu/100ml |
| Site H | effluent | EXTERNAL-08FEB22-358443 | TP | Phosphorus Total as P (Reading) | 0.13 | mg/L |
| Site H | effluent | EXTERNAL-08FEB22-358443 | COD | Chemical Oxygen Demand (Reading) | 4 | mg/L |
| Site H | effluent | EXTERNAL-08FEB22-358443 | TDS_EC | Electrical Conductivity @ 25 °C | 710 | uS/cm |
| Site H | effluent | EXTERNAL-08FEB22-358443 | TDS_EC | Total Dissolved Solids (TDS by EC Method | 430 | mg/L |
| Site H | effluent | EXTERNAL-08FEB22-358443 | FOG_AQ | Oil and Grease (Reading) | <0.15 | mg/L |
| Site H | effluent | EXTERNAL-08FEB22-358443 | NH3_N_500C | Ammoniacal Nitrogen (Reading) | 0.61 | mg/L |
| Site H | effluent | EXTERNAL-08FEB22-358443 | VHO_5L_TP | Total Ova Count / 5L | <1 | No./L |
| Site H | effluent | EXTERNAL-08FEB22-358443 | PHEN_TOTAL | Phenol Total | 0.0021 | mg/L |

**Table S3** Details of samples collected from selected WWTPs

| Total number of WWTPs | 8 (6 urban + 2 rural) = 8 WWTPs |
| --- | --- |
| Number of samples from influent and effluent across three sampling days from an urban WWTP for pharmaceuticals survey | 1 site × 3 days × 3 (triplicate samples) × 2 (influent & effluent) = 12 samples |
| Number of samples from influent & effluent from different WWTPs (urban & rural) for pharmaceuticals survey | 6 sites × 3 (triplicate samples) × 2 (influent & effluent) = 24 samples |
| Number of samples from influent & effluent & sludge from one an urban WWTP for radiopharmaceuticals survey | 1 site × 5 days × 3 (triplicate samples) × 3 (influent, effluent & sludge) = 30 samples |
| Overall number of samples | 66 samples |

**Table S4** Physico-chemical characteristics of targeted pharmaceuticals

| **No.** | **Analyte** | **Code** | **Pharmaceutical Class** | **Chemical Structure** | **MW (g/mol)** | **CAS No.** | **log *K*_ow_** | **p*K*_a_** |
| --- | --- | --- | --- | --- | --- | --- | --- | --- |
| 1 | Erythromycin Stearate | ERY | Antimicrobial (aminoglycoside) | 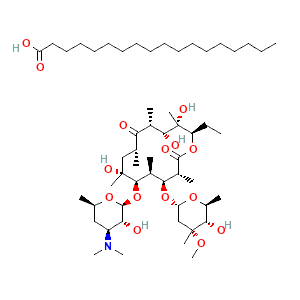 | 1018.4 | 643-22-1 | 3.06 (Erythromycin) | 8.80 (Erythromycin) |
| 2 | Metronidazole | MNZ | Antimicrobial (nitroimidazole) | 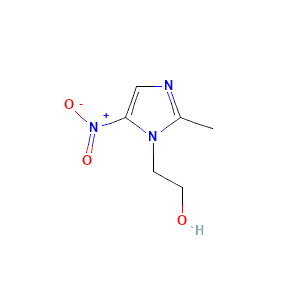 | 171.2 | 443-48-1 | -0.020 | 2.38 |
| 3 | Ofloxacin | OFX | Antimicrobial (quinolone) | 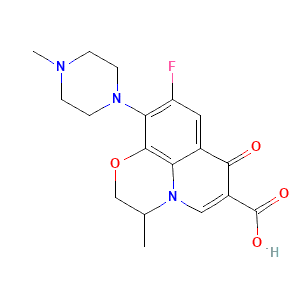 | 361.4 | 82419-36-1 | -0.39 | 8.31 |
| 4 | Trimethoprim | TMP | Antimicrobial (aminopyrimidine) | 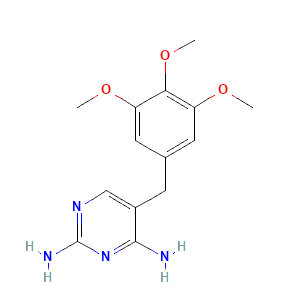 | 290.3 | 738-70-5 | 0.91 | 7.12 |
| 5 | Gliclazide | GLZ | Antihyperglycemic | 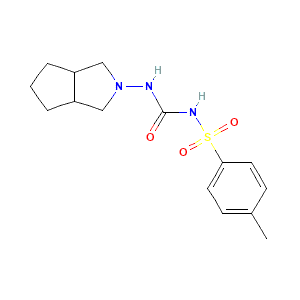 | 323.4 | 21187-98-4 | - | - |
| 6 | Metformin | MTF | Antihyperglycemic | 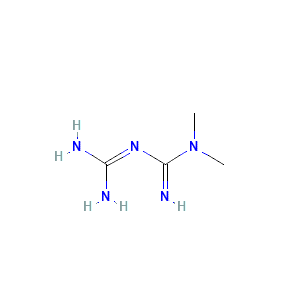 | 129.2 | 657-24-9 | -2.64 | 12.4 |
| 7 | Atorvastatin | ATS | Antilipemic (statin) | 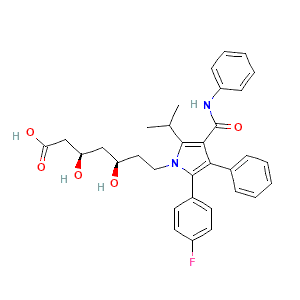 | 558.6 | 134523-00-5 | 6.36 | 4.46 |
| 8 | Simvastatin | SVS | Antilipemic (HMG-CoA reductase inhibitors (statins) ) | 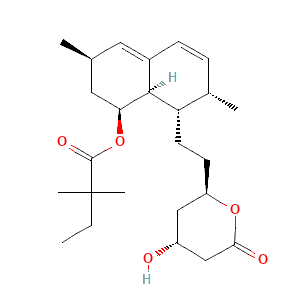 | 418.6 | 79902-63-9 | 4.68 | - |
| 9 | Atenolol | ATN | Antihypertensive, antiarrhythmic | 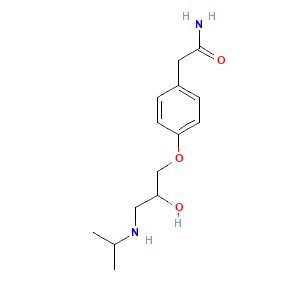 | 266.34 | 29122-68-7 | 0.16 | 9.58 |
| 10 | Captopril | CTP | Antihypertensive, antineoplastic | 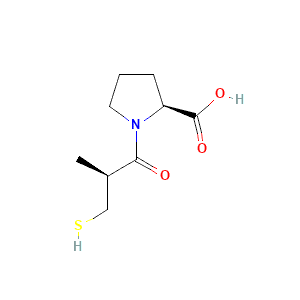 | 217.3 | 62571-86-2 | 0.34 | pKa1 = 3.70; pKa2 = 9.80 |
| 11 | Lisinopril | LSP | Antihypertensive | 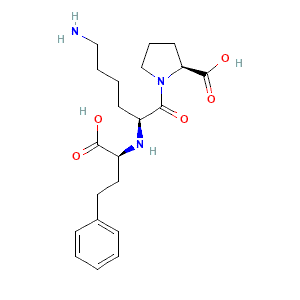 | 405.5 | 76547-98-3 | **-1.22** | 2.50 |
| 12 | Propranolol | PNL | Antihypertensive, antiarrhythmic, antianginal | 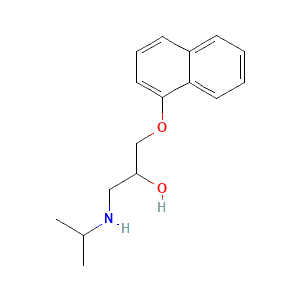 | 259.3 | 525-66-6 | - | 9.53 |
| 13 | Sildenafil | SDL | Urological agent, antihypertensive, antianginal | 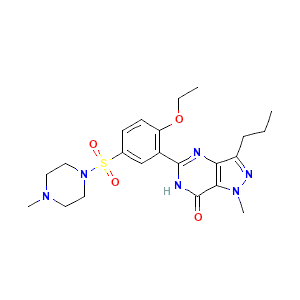 | 474.6 | 139755-83-2 | 2.75 | 5.99 |
| 14 | Amitriptyline | AMT | Antidepressants | 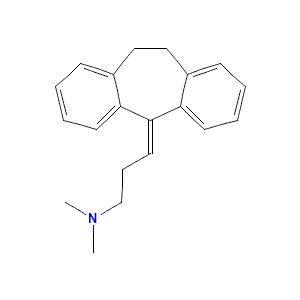 | 277.4 | 50-48-6 | 4.92 | 9.49 |
| 15 | Chlorpheniramine maleate | CPM | Antihistamine | 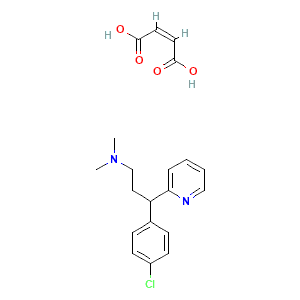 | 390.9 | 113-92-8 | - | 9.26 (Chlorpheniramine) |
| 16 | Diphenhydramine | DPH | Antihistamine | 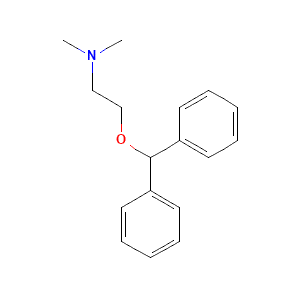 | 255.4 | 58-73-1 | 3.27 | 9.10 |
| 17 | Hyoscine butylbromide | HBB | Antispasmodic | 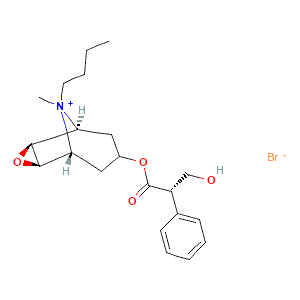 | 440.4 | 149-64-4 | - | - |
| 18 | Mefenamic Acid | MFA | Non-steroidal anti-inflammatory drug (NSAID) | 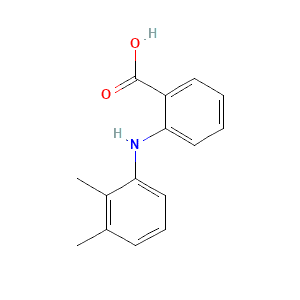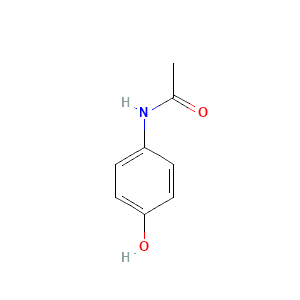   \|  \| \| --- \| | 241.3 | 61-68-7 | 3.26 * | 4.20 |
| 19 | Paracetamol | PCM | Analgesics, antipyretic |  | 151.2 | 103-90-2 | 0.46 | 9.38 |

**Table S5** MRM transitions of analytes in positive ion mode method

| **Compound** | **ESI** | **Precursor** | **Product** | **Frag (v)** | **CE (v)** |
| --- | --- | --- | --- | --- | --- |
| Metformin | (+) | 130 | 60 | 80 | 10 |
| Atenolol | (+) | 267.2 | 145.1 | 108 | 24 |
| Lisinopril | (+) | 406.2 | 84.1 | 136 | 30 |
| Trimethoprim | (+) | 291 | 230.2 | 146 | 21 |
| Paracetamol (Acetaminophen) | (+) | 152 | 110 | 90 | 15 |
| Metronidazole | (+) | 172.2 | 128.2 | 100 | 13 |
| Ofloxacin | (+) | 362.2 | 318.2 | 146 | 17 |
| Chlorpheniramine maleate | (+) | 275.1 | 230.4 | 150 | 8 |
| Hyoscine butylbromide | (+) | 360.3 | 138 | 100 | 30 |
| Propranolol | (+) | 260.2 | 56.1 | 146 | 29 |
| Diphenhydramine HCl | (+) | 256.2 | 167.2 | 68 | 12 |
| Erythromycin Stearate | (+) | 734.5 | 158 | 120 | 35 |
| Sildenafil | (+) | 474.6 | 58.2 | 191 | 49 |
| Amitriptyline | (+) | 278.2 | 91.1 | 108 | 29 |
| Captopril | (+) | 218.1 | 202.2 | 244 | 37 |
| Gliclazide | (+) | 324.1 | 91.1 | 103 | 37 |
| Atorvastatin | (+) | 559.3 | 250.2 | 181 | 49 |
| Mefenamic Acid | (+) | 242.1 | 224.2 | 103 | 13 |
| Simvastatin | (+) | 419.3 | 198.9 | 100 | 12 |

**Table S6** Mean percentage recovery of identified compounds in quality control samples from experimental (two spiking levels) and published data with comparable methodology

| **Pharmaceutical Compound** | **Mean Recovery % ± %RSD (n=3)** | | | | **Published Recovery (%)** | | |  |
| --- | --- | --- | --- | --- | --- | --- | --- | --- |
|  | **Influent** | | **Effluent** | | **Influent** | **Effluent** | **Reference** |  |
|  | **0.1 µg/L** | **0.5 µg/L** | **0.1 µg/L** | **0.5 µg/L** |  |  |  |  |
| Erythromycin | 95 ± 0.9 | 95 ± 1 | 97 ± 0.8 | 97 ± 0.7 | 130 (n=4) | 87-130 (n=4) | (Nieto-Juarez et al., 2021; Son et al., 2022) |  |
|  |  |  |  |  | 90-110 (n=7) | - |  |  |
| Metronidazole | 83 ± 1 | 88 ± 0.2 | 85 ± 0.5 | 91 ± 2 | 130-140 (n=4) | 110-115 (n=4) | (Nieto-Juarez et al., 2021) |  |
| Trimethoprim | 100 ± 0.7 | 95 ± 2 | 107 ± 2 | 97 ± 3 | 66-124 (n=4) | 79-101 (n=4) | (Nieto-Juarez et al., 2021; Son et al., 2022) |  |
|  |  |  |  |  | 100-110 (n=7) | - |  |  |
| Gliclazide | 86 ± 0.8 | 94 ± 0.4 | 87 ± 0.6 | 95 ± 1 | 65 - 105 (n=5) | - | (Iancu et al., 2021) |  |
| Metformin | 100 ± 2 | 85 ± 0.6 | 105 ± 0.8 | 86 ± 0.6 | 93 (n=3) | 93 (n=3) | (Oertel et al., 2018) |  |
|  |  |  |  |  |  |  |  |  |
| Atorvastatin | 82 ± 0.6 | 94 ± 2 | 83 ± 0.2 | 99 ± 1 | 100-150 (n=4) | 130-140 (n=4) | (Miao & Metcalfe, 2003; Nieto-Juarez et al., 2021) |  |
|  |  |  |  |  | 86 (n=3) | 64 (n=6) |  |  |
| Simvastatin | 82 ± 0.8 | 89 ± 0.8 | 84 ± 1 | 93 ± 1 | 84 (n=7) | 69 (n=6) | (Miao & Metcalfe, 2003) |  |
|  |  |  |  |  |  |  |  |  |
| Atenolol | 89 ± 2 | 81 ± 0.4 | 92 ± 0.5 | 86 ± 0.6 | 93 (n=5) | 110 (n=5) | (Al-Odaini et al., 2010; Al-Qaim et al., 2018; Son et al., 2022) |  |
|  |  |  |  |  | - | 82 (n=3) |  |  |
|  |  |  |  |  | 94-100 (n=7) | - |  |  |
| Captopril | 94 ± 1 | 94 ± 1 | 95 ± 0.5 | 97 ± 1 | 66 (n=5) | - | (Salgado et al., 2010) |  |
| Lisinopril | 83 ± 1 | 95 ± 1 | 86 ± 0.6 | 99 ± 2 | 80 (n=6) | - | (Tarcomnicu et al., 2011) |  |
| Propranolol | 95 ± 0.6 | 89 ± 0.4 | 97 ± 1 | 90 ± 0.2 | 84 (n=4) | - | (Iancu et al., 2021; Son et al., 2022) |  |
|  |  |  |  |  | 100-102 (n=7) | - |  |  |
| Sildenafil | 89 ± 0.5 | 97 ± 0.9 | 90 ± 1 | 98 ± 0.4 | 81-92 (n=7) | 81-92 (n=7) | (Hong et al., 2021; Son et al., 2022) |  |
|  |  |  |  |  | 100-120 (n=7) | - |  |  |
| Amitriptyline | 96 ± 1 | 91 ± 0.8 | 98 ± 0.3 | 92 ± 0.8 | 93-100 (n=6) | - | (Wang et al., 2024) |  |
| Chlorpheniramine | 89 ± 0.6 | 96 ± 0.5 | 91 ± 0.7 | 98 ± 0.1 | - | 52 (n=3) | (Al-Odaini et al., 2010) |  |
| Diphenhydramine | 94 ± 0.7 | 93 ± 0.5 | 96 ± 0.7 | 95 ± 0.3 | 97-100 (n=7) | - | (Son et al., 2022) |  |
| Hyoscine butyl bromide | 94 ± 0.6 | 96 ± 0.3 | 97 ± 1 | 96 ± 0.1 | - | 99-100 (n=3) | (W Ali & Gamal, 2013) |  |
|  |  |  |  |  |  |  |  |  |
| Mefenamic Acid | 84 ± 0.8 | 98 ± 1 | 86 ± 0.7 | 99 ± 1 | - | 86 (n=13) | (Al-Odaini et al., 2010) |  |
| Paracetamol (Acetaminophen) | 82 ± 0.6 | 84 ± 0.7 | 83 ± 1 | 87 ± 0.8 | 100-110 (n=7) | - | (Son et al., 2022) |  |
|  |  |  |  |  |  |  |  |  |
|  |  |  |  |  |  |  |  |  |
| Ofloxacin | 86 ± 1 | 85 ± 0.3 | 88 ± 0.6 | 86 ± 0.4 | 98-110 (n=7) | - | (Son et al., 2022) |  |

**Tab S7** Limits of detection (LOD) and Limits of quantification (LOQ) using Tandem MS analysis

| **Pharmaceutical Compound** | **LOD (ng/L)** | **LOQ (ng/L)** |
| --- | --- | --- |
| Erythromycin | 15.2 | 46.0 |
| Metronidazole | 6.6 | 20.0 |
| Trimethoprim | 13.6 | 41.3 |
| Gliclazide | 30.1 | 91.2 |
| Metformin | 22.7 | 68.8 |
| Atorvastatin | 17.1 | 51.8 |
| Simvastatin | 20.9 | 63.3 |
| Atenolol | 9.9 | 29.9 |
| Captopril | 35.9 | 108.9 |
| Lisinopril | 12.4 | 37.5 |
| Propranolol | 9.2 | 27.9 |
| Sildenafil | 17.3 | 52.5 |
| Amitriptyline | 42.4 | 128.4 |
| Chlorpheniramine | 15.5 | 47.0 |
| Diphenhydramine | 26.8 | 81.2 |
| Hyoscine butyl bromide | 19.9 | 60.2 |
| Mefenamic Acid | 13.1 | 39.6 |
| Paracetamol (Acetaminophen) | 7.0 | 21.3 |
| Ofloxacin | 22.7 | 68.9 |

**Table S8** Physico-radioactive characteristics of targeted radiopharmaceuticals (Mulas et al., 2019; PubChem, 2024)

| **No.** | **Radiopharmaceutical analyte** | **Code** | **Radiopharmaceutical use** | **Physical half-life (T½)** | **MW (g/mol)** | **CAS No.** | **Mean activity/Activity range (GBq)** |
| --- | --- | --- | --- | --- | --- | --- | --- |
| 1 | Iodine-131 | I-131 | Therapy:  Thyroid cancer  Hyperthyroidism | 8 days | 261.8 | 10043-66-0 | 1.1-7.4 0.19-0.80 |
| 2 | 18fluorine-fluorodeoxyglucose | 18F-FDG | Diagnosis radionuclides | ~ 2h  (110 minutes) | - | - | 0.34 |
| 3 | Technetium-99m | Tc-99m | Medical diagnosis radionuclides | 6 h | 98.9 | 14133-76-7 | 0.36 |
| 4 | Gallium-67 | 67-Ga | Diagnosis radionuclides | 78 h (3.26 days) | 69.7 | 7440-55-3 | 0.23 |

**Table S9** Detection status of pharmaceutical compounds across various locations. "D" indicates the presence of the compound (Detected), while "ND" denotes the absence (Not Detected).

| **No.** | **Pharmaceutical Compound** | **Compound Code** | **Location A** | **Location B** | **Location C** | **Location D** | **Location E** | **Location F** | **Location G** | **Location H** | **DF (%)** |
| --- | --- | --- | --- | --- | --- | --- | --- | --- | --- | --- | --- |
| 1 | Erythromycin | ERY | ND | ND | D | ND | ND | ND | ND | D | 25 |
| 2 | Metronidazole | MNZ | ND | D | ND | ND | ND | D | ND | ND | 25 |
| 3 | Trimethoprim | TMP | ND | ND | ND | ND | ND | ND | ND | ND | ND |
| 4 | Gliclazide | GLZ | D | ND | D | ND | ND | ND | ND | D | 38 |
| 5 | Metformin | MTF | D | D | ND | D | D | D | D | D | 88 |
| 6 | Atorvastatin | ATS | D | ND | D | D | ND | ND | ND | ND | 38 |
| 7 | Simvastatin | SVS | ND | ND | ND | ND | ND | ND | ND | ND | ND |
| 8 | Atenolol | ATN | D | D | D | D | D | D | D | D | 100 |
| 9 | Captopril | CTP | ND | ND | ND | ND | D | ND | ND | ND | 13 |
| 10 | Lisinopril | LSP | D | D | D | D | D | D | D | D | 100 |
| 11 | Propranolol | PNL | ND | ND | ND | ND | ND | ND | ND | ND | ND |
| 12 | Sildenafil | SDF | ND | ND | ND | ND | ND | ND | ND | D | 13 |
| 13 | Amitriptyline | AMT | ND | ND | ND | ND | D | ND | ND | ND | 13 |
| 14 | Chlorpheniramine maleate | CPM | ND | ND | ND | ND | ND | ND | ND | ND | ND |
| 15 | Diphenhydramine | DPH | ND | ND | ND | ND | D | ND | ND | ND | 13 |
| 16 | Hyoscine butyl bromide | HBB | ND | ND | ND | ND | ND | ND | ND | ND | ND |
| 17 | Mefenamic Acid | MFA | D | D | D | D | D | D | D | D | 100 |
| 18 | Paracetamol (Acetaminophen) | PCM | D | D | D | D | D | D | ND | D | 88 |
| 19 | Ofloxacin | OFX | ND | ND | ND | ND | ND | ND | ND | ND | ND |

**References:**

Al-Odaini, N. A., Zakaria, M. P., Yaziz, M. I., & Surif, S. (2010). Multi-residue analytical method for human pharmaceuticals and synthetic hormones in river water and sewage effluents by solid-phase extraction and liquid chromatography–tandem mass spectrometry. *Journal of Chromatography A*, *1217*(44), 6791-6806. <https://doi.org/10.1016/j.chroma.2010.08.033>

Al-Qaim, F. F., Mussa, Z. H., & Yuzir, A. (2018). Development and validation of a comprehensive solid-phase extraction method followed by LC-TOF/MS for the analysis of eighteen pharmaceuticals in influent and effluent of sewage treatment plants. *Analytical and Bioanalytical Chemistry*, *410*(20), 4829-4846. <https://doi.org/10.1007/s00216-018-1120-9>

Hong, Y., Lee, I., Tae, B., Lee, W., Pan, S.-Y., Snyder, S. W., & Kim, H. (2021). Contribution of sewage to occurrence of phosphodiesterase-5 inhibitors in natural water. *Scientific reports*, *11*(1), 9470-9470. <https://doi.org/10.1038/s41598-021-89028-3>

Iancu, V.-I., Scutariu, R.-E., Chiriac, F.-L., & Radu, G.-L. (2021). Sensitive detection of antidiabetic compounds and one degradation product in wastewater samples by a new SPE-LC-MS/MS method. *Journal of Environmental Science and Health, Part A*, *56*(3), 310-323. <https://doi.org/10.1080/10934529.2021.1873671>

Miao, X.-S., & Metcalfe, C. D. (2003). Determination of cholesterol-lowering statin drugs in aqueous samples using liquid chromatography–electrospray ionization tandem mass spectrometry. *Journal of Chromatography A*, *998*(1), 133-141. <https://doi.org/10.1016/S0021-9673(03)00645-9>

Mulas, D., Camacho, A., Garbayo, A., Devesa, R., & Duch, M. A. (2019). Medically-derived radionuclides levels in seven heterogeneous urban wastewater treatment plants: The role of operating conditions and catchment area. *Sci Total Environ*, *663*, 818-829. <https://doi.org/10.1016/j.scitotenv.2019.01.349>

Nieto-Juarez, J. I., Torres-Palma, R. A., Botero-Coy, A. M., & Hernandez, F. (2021). Pharmaceuticals and environmental risk assessment in municipal wastewater treatment plants and rivers from Peru. *Environment International*, *155*, 106674. <https://doi.org/10.1016/j.envint.2021.106674>

Oertel, R., Baldauf, J., & Rossmann, J. (2018). Development and validation of a hydrophilic interaction liquid chromatography-tandem mass spectrometry method for the quantification of the antidiabetic drug metformin and six others pharmaceuticals in wastewater. *Journal of Chromatography A*, *1556*, 73-80. <https://doi.org/10.1016/j.chroma.2018.04.068>

PubChem. (2024). *Compounds summary (explore chemistry)*. National Library of Medicine. Retrieved 15 October 2024 from <https://pubchem.ncbi.nlm.nih.gov/>

Salgado, R., Noronha, J. P., Oehmen, A., Carvalho, G., & Reis, M. A. M. (2010). Analysis of 65 pharmaceuticals and personal care products in 5 wastewater treatment plants in Portugal using a simplified analytical methodology. *Water Science and Technology*, *62*(12), 2862-2871. <https://doi.org/10.2166/wst.2010.985>

Son, D.-J., Kim, C.-S., Park, J.-W., Lee, J.-H., Lee, S.-H., Shin, S.-K., & Jeong, D.-H. (2022). Fate evaluation of pharmaceuticals in solid and liquid phases at biological process of full-scale municipal wastewater treatment plants. *Journal of Water Process Engineering*, *46*, 102538. <https://doi.org/10.1016/j.jwpe.2021.102538>

Tarcomnicu, I., van Nuijs, A. L. N., Simons, W., Bervoets, L., Blust, R., Jorens, P. G., Neels, H., & Covaci, A. (2011). Simultaneous determination of 15 top-prescribed pharmaceuticals and their metabolites in influent wastewater by reversed-phase liquid chromatography coupled to tandem mass spectrometry. *Talanta*, *83*(3), 795-803. <https://doi.org/10.1016/j.talanta.2010.10.045>

W Ali, N., & Gamal, M. (2013). LC-MS as a Stability-Indicating Method for Analysis of Hyoscine N-Butyl Bromide under Stress Degradation Conditions with Identification of Degradation Products. *Pharmaceutica analytica acta*, *s7*. <https://doi.org/10.4172/2153-2435.S7-006>

Wang, A., Zhang, J., Hu, L., Yu, Z., Lai, S., Liu, Y., Mai, Z., & Xu, M. (2024). Trace analysis of 47 psychotropic medications in environmental samples by ultra-performance liquid chromatography tandem mass spectrometry (UPLC-MS/MS). *Journal of Chromatography A*, *1715*, 464627. <https://doi.org/10.1016/j.chroma.2023.464627>
